# Novel prognostic marker TGFBI affects the migration and invasion function of ovarian cancer cells and activates the integrin αvβ3-PI3K-Akt signaling pathway

**DOI:** 10.1186/s13048-024-01377-5

**Published:** 2024-02-23

**Authors:** Hao Wang, Yin-hai Xu, Yi Guo

**Affiliations:** 1 School of Medical Technology, Xuzhou Medical University, Xuzhou, Jiangsu China; 2 Xuzhou Key Laboratory of Laboratory Diagnostics, Xuzhou Medical University, Xuzhou, Jiangsu China; 3 Department of Laboratory Medicine, Affiliated Hospital of Xuzhou Medical University, Xuzhou, Jiangsu China; 4 Department of Laboratory Medicine, The Second Affiliated Hospital of Xuzhou Medical University, Xuzhou, Jiangsu China; 5 School of Medical Technology, Xuzhou Medical University, Xuzhou, Jiangsu 221004 China; 6 Department of Laboratory Medicine, The Second Affiliated Hospital of Xuzhou Medical University, Xuzhou, Jiangsu 221006 China

**Keywords:** Ovarian cancer, TGFBI, Prognosis, Migration, Invasion, Metastasis, PI3K-Akt signaling pathway, Integrin αvβ3

## Abstract

**Background:**

Individual patients with ovarian cancer show remarkably different prognosis. Present prognostic models for ovarian cancer mainly focus on clinico-pathological parameters, so quantifiable prognostic markers at molecular level are urgently needed. Platelets contribute to ovarian cancer progression, but have not been considered as biomarkers likely due to their instability. Here, we aimed to search for a stable prognostic marker from platelet-treated ovarian cancer cells, and explore its functions and mechanisms.

**Methods:**

Microarrays analysis was done with platelet-treated SKOV-3 ovarian cancer cells. Relevant studies were searched in the Gene Expression Omnibus (GEO) database. The candidate genes were determined by differentially expressed genes (DEGs), Venn diagram drawing, protein-protein interaction (PPI) network, Cox proportional hazards model and Kaplan-Meier analysis. The expression of TGFBI in clinical samples was assessed by immunehistochemical staining (IHC), and the association of TGFBI levels with the clinic-pathological characteristics and prognosis in ovarian cancer patients was evaluated by univariate and multivariate analysis. The functions of TGFBI were predicted using data from TCGA, and validated by in vitro and in vivo experiments. The mechanism exploration was performed based on proteomic analysis, molecular docking and intervention study.

**Results:**

TGFBI was significantly higher expressed in the platelet-treated ovarian cancer cells. An analysis of bioinformatics data revealed that increased expression of TGFBI led to significant decrease of overall survival (OS), progression-free survival (PFS) and post-progression survival (PPS) in ovarian cancer patients. Tissue microarray results showed that TGFBI was an independent factor for ovarian cancer, and TGFBI expression predict poor prognosis. Functionally, TGFBI affected the migration and invasion of ovarian cancer cells by regulation of epithelial mesenchymal transition (EMT) markers (CDH1 and CDH2) and extracellular matrix (ECM) degradation proteins (MMP-2). Mechanistically, TGFBI phosphorylated PI3K and Akt by combining integrin αvβ3.

**Conclusions:**

We found out TGFBI as a novel prognostic indicator for ovarian cancer patients. TGFBI could promote metastasis in ovarian cancer by EMT induction and ECM remodeling, which might be associated with the activation of integrin αvβ3-PI3K-Akt signaling pathway.

**Supplementary Information:**

The online version contains supplementary material available at 10.1186/s13048-024-01377-5.

## Introduction

Ovarian cancer is a gynecologic malignancy with high mortality worldwide, resulting in 210,000 deaths per year [1]. In western countries, ovarian cancer is the most fatal gynecologic neoplasms, with a 5-year cause-specific survival of 47% on average [2]. The change of ovarian cancer mortality was 0.76% per year from 1990 to 2019 in China, which exhibited a sustained growth trend as well [3]. Although the ovarian cancer was characterized by metastasis, rapid progression and an **overall** bad prognosis, individual patients showed remarkably different outcomes during disease course. While some patients could be maintained on chemotherapy for over 5 years and developed into chronic disease, others were intrinsically chemo-resistant or rapidly transformed into chemo-resistant after an initial period of chemo-sensitivity, [4]. Precise determination of ovarian cancer patients with poor outcomes would facilitate personalized treatment strategies or timely inclusion into clinical trials. Present prognostic index for ovarian cancer mainly focused on clinico-pathological parameters, like age at diagnosis, tumor stage and histological subtype. Nonetheless, the occurrence and progression of ovarian cancer involved complex cell-cell interactions and molecular regulatory mechanisms, thus qualitative predictors were limited by their efficiency and consistency among patients [5]. Alternatively, exploration of quantifiable prognostic biomarkers at cellular and molecular levels would further improve risk stratification and prognostic prediction for ovarian cancer patients [6].

The platelet count in peripheral blood has impact on the prognosis of patients with ovarian cancer. An early study of 70 patients in Israel found that ovarian cancer patients with thrombocytosis (>400 × 10^9^/L) had worse overall survival (OS) [7]. In a large study of 816 patients in China, thrombocytosis was associated with significantly increased hazards of disease progression and death in ovarian cancer [8]. However, there has been no clinical guidelines referring the platelet count as a prognostic marker for ovarian cancer, because the number of platelet is susceptible to various factors, such as thromboembolism, acute inflammation and stress response [9].

Previously, we have published a relevant experimental research, revealing that platelets increased the invasive ability of ovarian cancer cells by TGF-β signaling pathway [10]. In the study, we co-incubated the ovarian cancer cells with platelets, and found out a significant alteration in their gene expression profiles, which have been well recognized by scholars worldwide [11, 12]. Therefore, we next planned to select out abnormally expressed genes as stable molecular predictors based on the platelet-treated ovarian cancer cell model. To avoid false findings generated by limited samples size in a single independent study, integrated analysis with collective datasets was recommended. Unlike other publicly available databases mainly including tumor samples and normal tissues, Gene Expression Omnibus (GEO) also contains tumor cells with platelet-treatment. Thus, comprehensive analysis was carried out between our microarray results and GEO sample series.

Firstly, we explored differentially expressed genes (DEGs) using data from our microarray and GEO. This was followed by Gene Ontology (GO) analysis and Kyoto Encyclopedia of Genes and Genomes (KEGG) pathways enrichment. Next, core genes were identified by protein-protein interaction (PPI) network and Cox proportional hazards regression analysis, and those showing significant hazard ratios for ovarian cancer patients in The Cancer Genome Atlas (TCGA) were considered as key genes. Among them, those significantly affecting prognosis of patients in Kaplan-Meier plotter database were selected as candidate genes. Finally, the functions of candidate genes were predicted using data from TCGA by R software, and validated by in vitro and in vivo experiments. The mechanism exploration was performed based on proteomic analysis, molecular docking and intervention study.

In this study, we aimed to: (1) screen out the new biomarkers at molecular level from platelet-treated ovarian cancer cell model for ovarian cancer prognosis prediction; (2) performed an initial exploration of their functions and mechanisms to provide theoretical basis for their clinical translation.

## Results

### The included items from GEO database

Fifty-one studies were listed by GEO repository according to our search strategy. After screening titles and abstracts, 3 studies met the inclusion criteria. For others, 2 studies did not treat ovarian cancer cells with platelets in accordance with the grouping principles mentioned in 2.5, 44 studies only presented a single sample in the GSE datasets, and 2 studies performed RNA sequencing on platelets. Two of the remaining three studies were excluded, since GSE155547 contained mixed-cell samples and human ovarian cancer cells were treated with mouse platelets in GSE 95,275. Therefore, GSE155546 were finally included and analyzed with our gene chip data [13].

In the GSE155546, G164 malignant cells, a kind of high-grade serous ovarian cancer cell line, were treated with or without platelets for 24 h (named P and C, respectively), and each group had 3 samples for biological repeats. Illumina HiSeq 4000 (GPL20301) was applied for the high-throughput RNA sequencing for ovarian cancer cells.

### Gene expression changes in GSE155546 and our own dataset (named P vs. C)

The volcano plot showed that a total of 4553 mRNAs were differentially expressed between the two groups from our own microarray analysis with a significant level of p value < 0.05 and absolute fold change ≧ 1.5, including 1930 up-regulated and 2405 down-regulated genes (Figure S1 B). Whereas, only 260 genes exhibited significantly differential expression in GSE155546, of which 152 ones were highly expressed and 108 were down-expression (Figure S1 A). The top 50 DEGs were determined in the two sets respectively, and showed as heat maps (Figure S1 C and D).

### GO analysis and KEGG enrichment for DEGs

GO annotations for DEGs from GSE155546 and our own dataset were categorized into three hierarchical frameworks as cellular component (CC), molecular function (MF) and biological process (BP) (Figure S2 A and B). Notably, DEGs located in the extracellular matrix (ECM), related to ECM structural constituent and involved in extracellular matrix organization were found in both datasets. This result strongly indicated that platelets could alter matrisome in ovarian cancer cells. KEGG cluster also presented that ECM-related pathways were abnormally activated in platelet-treated ovarian cancer cells (Figure S2 C and D). Besides, terms related to development, such as heart and vasculature development, were commonly enriched in the two sets (Figure S2 A), which suggested that platelets could reverse development procedure and induce mesenchymal transition in ovarian cancer cells [10, 14]. GO enrichment and KEGG pathway analysis provided matrisome as research direction for us to explore key genes in the ovarian cancer progression primed by platelets.

### Core genes selection

**Fig. 1:**
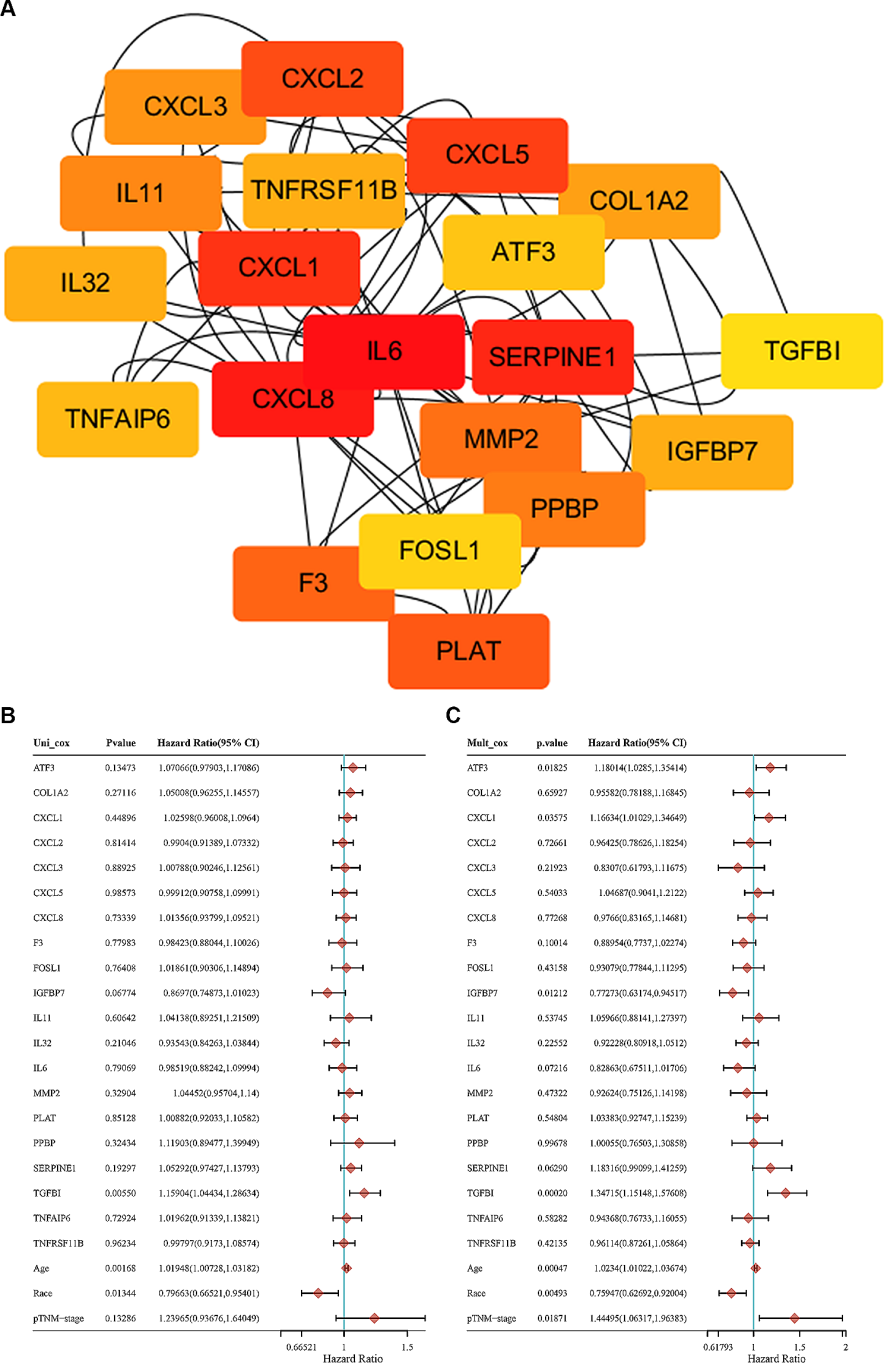
Brief description of process for key genes selection. **A** The core genes from PPI network. **B** Univariate analysis of prognostic factors in ovarian cancer patients from TCGA database. **C** Multivariate analysis of prognostic factors in ovarian cancer patients from TCGA database. *PPI* Integration of protein-protein interaction, *TCGA* The Cancer Genome Atlas

Altogether, we found 88 overlapping DEGs between the two datasets (Figure S3 A). Among them, 35 genes were commonly up-regulated, 13 genes were commonly down-regulated (Figure S3 B). Moreover, 40 genes showed opposite directions of change: 29 genes were up-regulated in GSE155546 but down-regulated in P vs. C dataset, while 13 genes were down-regulated in GSE155546 but up-regulated in P vs. C dataset (Figure S3 B). The specific gene names and their expression in the platelet-treated groups and control groups were represented as heat maps (Figure S3 C and D). PPI network analysis showed that there were 42 nodes and 116 edges among the common DEGs (Figure S4 A). The top 20 genes rated by the algorithm in Cytoscape were SERPINE1, COL1A2, MMP2, IL6, F3, IL32, CXCL5, CXCL2, CXCL3, FOSL1, IL11, TNFAIP6, CXCL8, ATF, CXCL1, PPBP, PLAT, IGFBP7, TNFRSF11B and TGFBI (Fig. 1A and Figure S4 B), and they were selected as core genes for further analysis. It is worth noting that COL1A2, MMP2 and TGFBI were the three genes related to ECM.

### Candidate genes determination

**Fig. 2:**
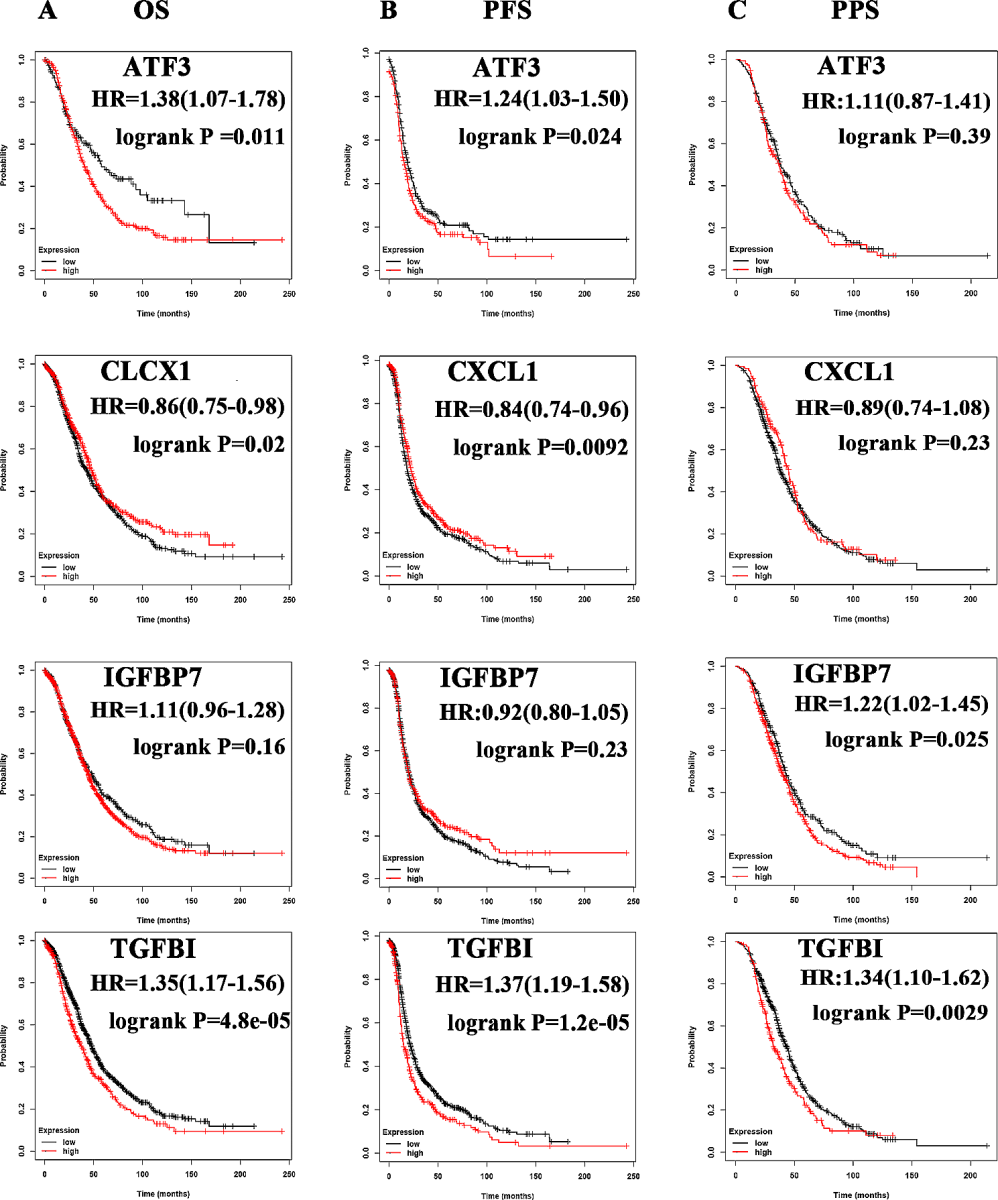
Candidate genes selection using survival analysis. **A** Kaplan-Meier plot of OS analysis for key genes. **B** Kaplan-Meier plot of PFS analysis for key genes. **C** Kaplan-Meier plot of PPS analysis for key genes. *OS* overall survival, *PFS* progress-free survival, *PPS* post-progression survival

Cox proportional-hazard model was used to test the prognostic value of the 20 core genes on 374 patients from TCGA database. Univariate analysis indicated that TGFBI was a prognostic predictor for ovarian cancer patients (HR = 1.16, 95% CI: 1.04–1.29, *p* < 0.01) (Fig. 1B and Figure S5A), and it was still proved to be an independent adverse factor in the multivariate analysis (HR = 1.35, 95% CI: 1.15–1.58, *p* < 0.01) (Fig. 1C and Figure S5B). In addition, high expression of AFT3 and CXCL1 showed worse prognosis in ovarian cancer, while IGFBP7 behaved as a protective predictor according to multivariable analysis (Figure S5 B). Therefore, TGFBI, AFT3, CXCL1 and IGFBP7 were screen out as key genes. The nomogram was plotted to predict 1-, 3-, and 5-year OS of a patient with ovarian cancer using the total points calculated by her key genes, tumor stage, age and race. The C-index of this algorithm reached to 0.65(95%CI: 0.612-1, *p* < 0.01) (Figure S5 C). The calibration curves of the nomogram showed satisfactory consistency between predicted and actual results (Figure S5 D). The prognostic values of the 4 key genes were further certified using a cohort of ovarian cancer patients from Kaplan-Meier Plotter database. Patients were spilt by median gene expression, and only high expression of TGFBI led to significant decrease of three prognostic index [OS: HR = 1.35, 95%CI: 1.17–1.56, *p* < 0.01; PFS: HR = 1.37, 95%CI: 1.19–1.58, *p* < 0.01; PPS: HR = 1.34, 95%CI: 1.10–1.62, *p* < 0.01]. Other details could be seen in the Fig. 2. Thus, TGFBI was prioritized as candidate gene for function exploration.

### Validation of the prognostic value of TGFBI at the protein level

**Fig. 3:**
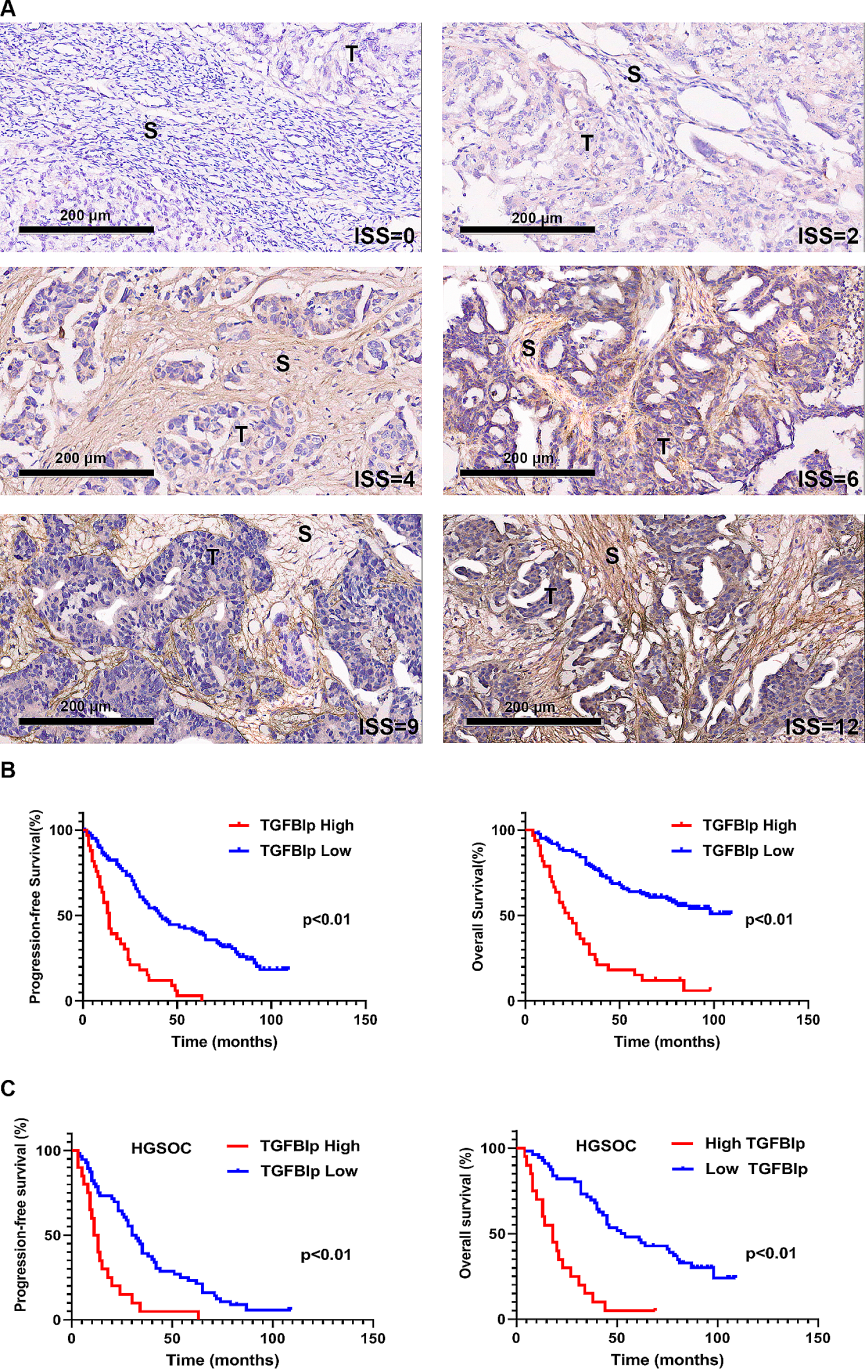
The relations between intratumoral TGFBIp expression and PFS and OS of patients with ovarian cancer. **A** Representative IHC images of TGFBIp in the specimens from patients with ovarian cancer. **B** Kaplan-Meier survival curves of patients with ovarian cancer based on high versus low levels of TGFBIp. **C** Kaplan-Meier survival curves of patients with HGSOC based on high versus low levels of TGFBIp. *T* tumor, *S* stroma, *IHC* immunohistochemical, *PFS* progress-free survival, *OS* overall survival, *HGSOC* high grade serous ovarian carcinomas

IHC staining was used to analyze the expression of TGFBI encoded protein (TGFBIp) in a cohort of individuals with ovarian cancer. This revealed that varying degrees of TGFBIp expression were seen in the ovarian tumor tissues (Fig. 3A). Using X-title software, the optimal cut-off value of the ISS of TGFBIp was 6 both for PFS and OS. Kaplan-Meier survival analysis further indicated that TGFBIp is negatively associated with prognosis-patients who exhibit high intratumoural TGFBIp expression survived shorter than those with low TGFBIp (Fig. 3B), especially for those with high grade serous ovarian carcinomas (HGSOC) (Fig. 3C).

The effects of different variables on PFS and OS of ovarian cancer were analyzed by univariate and multivariate analysis. Univariate analysis exhibited that tumor size, tumor grade, FIGO stage, TGFBIp expression and Ki67 expression significantly affected survival time (Table 1). Following the iterative analysis of the multivariate model, the TGFBIp expression proved to be a strong prognostic predictor (*p* < 0.01), as shown in Table 2. Moreover, TGFBIp was also an independent risk factor for PFS (HR = 2.68, 95%CI: 1.54–4.66, *p* < 0.01) and OS (HR = 3.67, 95%CI: 1.98–6.82, *p* < 0.01) in HGSOC patients, and details could be seen in the Tables 3 and 4.

**Table 1:** Basic clinical information of patients with ovarian cancer in tissue microarray

Characteristics	n (%)
Age	
**< 60**	114
**≥ 60**	44
Tumor size	
**< 5 cm**	20
**5-10 cm**	42
**>10 cm**	96
Histology	
**high grade serous carcinomas**	76
**low grade serous carcinomas**	35
**mucinous carcinoma**	19
**endometrioid carcinoma**	16
**others**	12
Grade	
**G1**	21
**G2**	38
**G3**	90
**G4**	9
FIGO stage	
**I**	9
**II**	37
**III**	79
**IV**	33

Others: clear cell carcinoma: 8, dysgerminoma: 2, yolk sac tumor:1, Brenner tumor: 1

**Table 2:** Univariate and multivariate analysis of the factors correlated with progression - free survival (PFS) of ovarian cancer patients

Variables	Univariate analysis					Multivariate analysis		
	HR	95%CI	p value			HR	95%CI	p value
Age (≥ 60)	1.74	1.20–2.54	< 0.01			0.74	0.49–1.13	0.16
Tumor size								
**< 5 cm**	1					1		
**5-10 cm**	0.72	0.49–1.08	0.11					
**>10 cm**	0.44	0.30–0.65	< 0.01			2.05	1.33–3.17	< 0.01
Histology								
**serous carcinomas**	1					1		
**mucinous carcinoma**	0.76	0.43–1.31	0.32					
**endometrioid carcinoma**	0.63	0.34–1.18	0.15					
**others**	0.33	0.13–0.80	0.02			0.58	0.23–1.53	0.28
Grade								
**G1**	1					1		
**G2**	1.47	0.96–2.27	0.08					
**G3**	1.52	1.06–2.19	0.02			0.77	0.50–1.18	0.22
**G4**	4.74	2.31–9.73	< 0.01			1.87	0.74–4.70	0.19
FIGO stage								
**I**	1					1		
**II**	3.98	2.34–6.76	< 0.01			3.89	0.51–29.59	0.19
**III**	1.91	1.33–2.73	< 0.01			19.69	2.65-146.25	< 0.01
**IV**	4.10	2.65–6.32	< 0.01			38.21	4.83-301.96	< 0.01
TGFBIp expression	3.72	2.44–5.68	< 0.01			3.13	1.97–4.99	< 0.01
Ki67 expression	1.52	1.00-2.30	0.04			0.90	0.57–1.43	0.65
EGFR expression	0.81	0.57–1.15	0.23					
PDL1 expression	1.20	0.83–1.71	0.34					

**Table 3:** Univariate and multivariate analysis of the factors correlated with overall survival (OS) of ovarian cancer patients

Variables	Univariate analysis					Multivariate analysis		
	HR	95%CI	p value			HR	95%CI	p value
Age (≥ 60)	0.58	0.37–0.90	0.01			0.78	0.49–1.25	0.30
Tumor size								
**< 5 cm**	1					1		
**5-10 cm**	0.48	0.28–0.83	< 0.01			2.91	1.15–6.74	0.04
**>10 cm**	4.94	2.81–8.71	< 0.01			4.65	3.22–8.34	< 0.01
Histology								
**serous carcinomas**	1					1		
**mucinous carcinoma**	0.54	0.25–1.17	0.12					
**endometrioid carcinoma**	0.44	0.18–1.09	0.08					
**others**	0.37	0.11–1.17	0.09			0.58	0.23–1.53	0.28
Grade								
**G1**	1					1		
**G2**	0.59	0.33–1.03	0.06					
**G3**	1.76	1.12–2.77	0.01			0.89	0.51–1.56	0.69
**G4**	4.30	2.13–8.67	< 0.01			1.44	0.56–3.69	0.45
FIGO stage								
**I**	1					1		
**II**	0.11	0.04–0.30	< 0.01			2.21	0.71–8.19	0.34
**III**	2.32	1.16–4.03	< 0.01			8.57	2.65–83.34	< 0.01
**IV**	5.74	3.60–9.17	< 0.01			23.33	5.61–124.79	< 0.01
TGFBIp expression	4.41	2.79–6.97	< 0.01			3.65	2.22–5.99	< 0.01
Ki67 expression	1.86	1.17–2.97	< 0.01			1.16	0.71–1.89	0.56
EGFR expression	0.74	0.48–1.13	0.16					
PDL1 expression	1.39	0.89–2.17	0.15					

**Table 4:** Univariate and multivariate analysis of the factors correlated with progression - free survival (PFS) of HGSOC patients

Variables	Univariate analysis					Multivariate analysis		
	HR	95%CI	p value			HR	95%CI	p value
Age (≥ 60)	1.27	0.77–2.11	0.35					
Tumor size								
**< 5 cm**	1							
**5-10 cm**	0.81	0.31–2.12	0.25					
**>10 cm**	1.93	0.82–4.54	0.13					
FIGO stage								
**I+II**	1					1		
**III+IV**	3.64	1.44–9.21	< 0.01			3.17	1.24–8.10	0.01
TGFBIp expression	3.06	1.77–5.32	< 0.01			2.68	1.54–4.66	< 0.01
Ki67 expression	1.40	0.84–2.31	0.19					
EGFR expression	0.67	0.42–1.08	0.10					
PDL1 expression	1.29	0.78–2.12	0.32					

*Note*: HGSOC: high grade serous ovarian carcinoma

**Table 5:** Univariate and multivariate analysis of the factors correlated with overall survival (OS) of HGSOC patients

Variables	Univariate analysis					Multivariate analysis		
	HR	95%CI	p value			HR	95%CI	p value
Age (≥ 60)	1.39	0.80–2.49	0.24					
Tumor size								
**< 5 cm**	1					1		
**5-10 cm**	4.20	0.93–18.92	0.62					
**>10 cm**	11.34	2.53–50.79	< 0.01			11.76	2.48–55.87	< 0.01
FIGO stage								
**I+II**	1					1		
**III+IV**	10.73	1.48–77.64	0.02			7.91	1.07–86.66	0.04
TGFBIp expression	4.92	2.67–9.04	< 0.01			3.67	1.98–6.82	< 0.01
Ki67 expression	1.53	0.90–2.61	0.12					
EGFR expression	0.68	0.41–1.13	0.14					
PDL1 expression	1.28	0.74–2.21	0.37					

*Note*: HGSOC: high grade serous ovarian carcinoma

### Function prediction for the candidate gene TGFBI

Spearman correlation analysis was applied to explore the potential functions of TGFBI, which was up-regulated by platelet-treatment. There was a significant positive association between the expression of TGFBI and TGF-β (ρ = 0.57, 95%CI: 0.50–0.64, *p* < 0.01) (Figure S6 A). A significant positive correlation between TGFBI levels and the expression of epithelial to mesenchymal transition (EMT) markers was also observed (ρ = 0.65, 95%CI: 0.59–0.71, *p* < 0.01) (Figure S6 B), which indicated that TGFBI might induce ovarian cancer cells to lose adhesive epithelial phenotype and acquire movable features of mesenchyme. ECM-related genes (ρ = 0.56, 95%CI: 0.48–0.62, *p* < 0.01) and degradation of ECM (ρ = 0.61, 95%CI: 0.54–0.67, *p* < 0.01) were also significantly positively correlated with TGFBI expression (Figure S6 C and D), suggesting that TGFBI might regulate ECM remodeling in ovarian cancer microenvironment.

### Molecular validations of the TGFBI functions

**Fig. 4:**
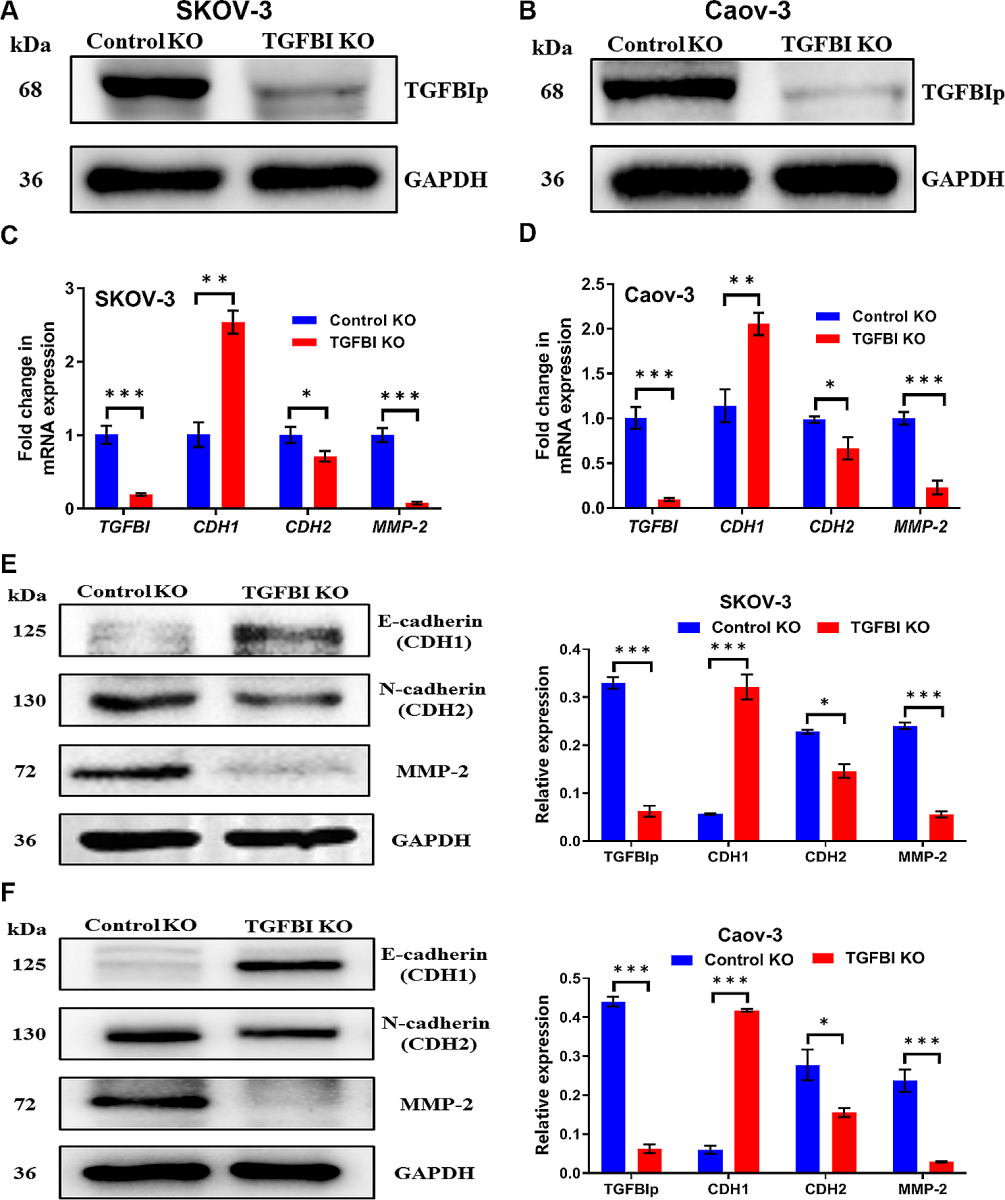
Molecular validations of TGFBI functions. **A**, **B** TGFBI KO confirmed by WB in SKOV-3 cell line and Caov-3 cell line. **C**, **D** Analysis of the expression changes of EMT markers (CDH1, CDH2 and MMP-2) in TGFBI KO cell lines at mRNA level, values were normalized to GAPDH. **E**, **F** Analysis of the expression changes of EMT markers (CDH1, CDH2 and MMP-2) in TGFBI KO cell lines at protein level. *KO* knock out, *WB* western blot, *MMP-2* matrix metallopeptidase 2, *GAPDH* glyceraldehyde-3-phosphate dehydrogenase. *p < 0.05, **p < 0.01, ***p < 0.001

To verify the bioinformatics prediction of potential functions for the selected candidate gene, we knocked out (KO) the expression of TGFBI in the SKOV-3 and Caov-3 cell lines using CRISPR-Cas9 lentivirus (Fig. 4A and B). Significantly increased expression of CDH1 (E-cadherin) and significantly decreased expression of CDH2 (N-cadherin) were demonstrated by qRT-PCR and WB following the TGFBI KO (Fig. 4C, D, E and F). The E-cadherin expressions in TGFBI KO cells and their controls were also detected using immunofluorescence, details could be seen in the Figure S8. These results suggested that TGFBI had the potential to affect the motility of ovarian cancer cells via EMT regulation. Besides, cells treated with lentivirus containing TGFBI-sgRNA plasmid significantly down-regulated the MMP-2 expression both at the mRNA and protein expression levels in comparison with the scramble control cells (Fig. 3C, D, E and F), indicating that TGFBI could facilitate tissue barrier destruction by degrading ECM.

### Cellular validations of the TGFBI functions

**Fig. 5:**
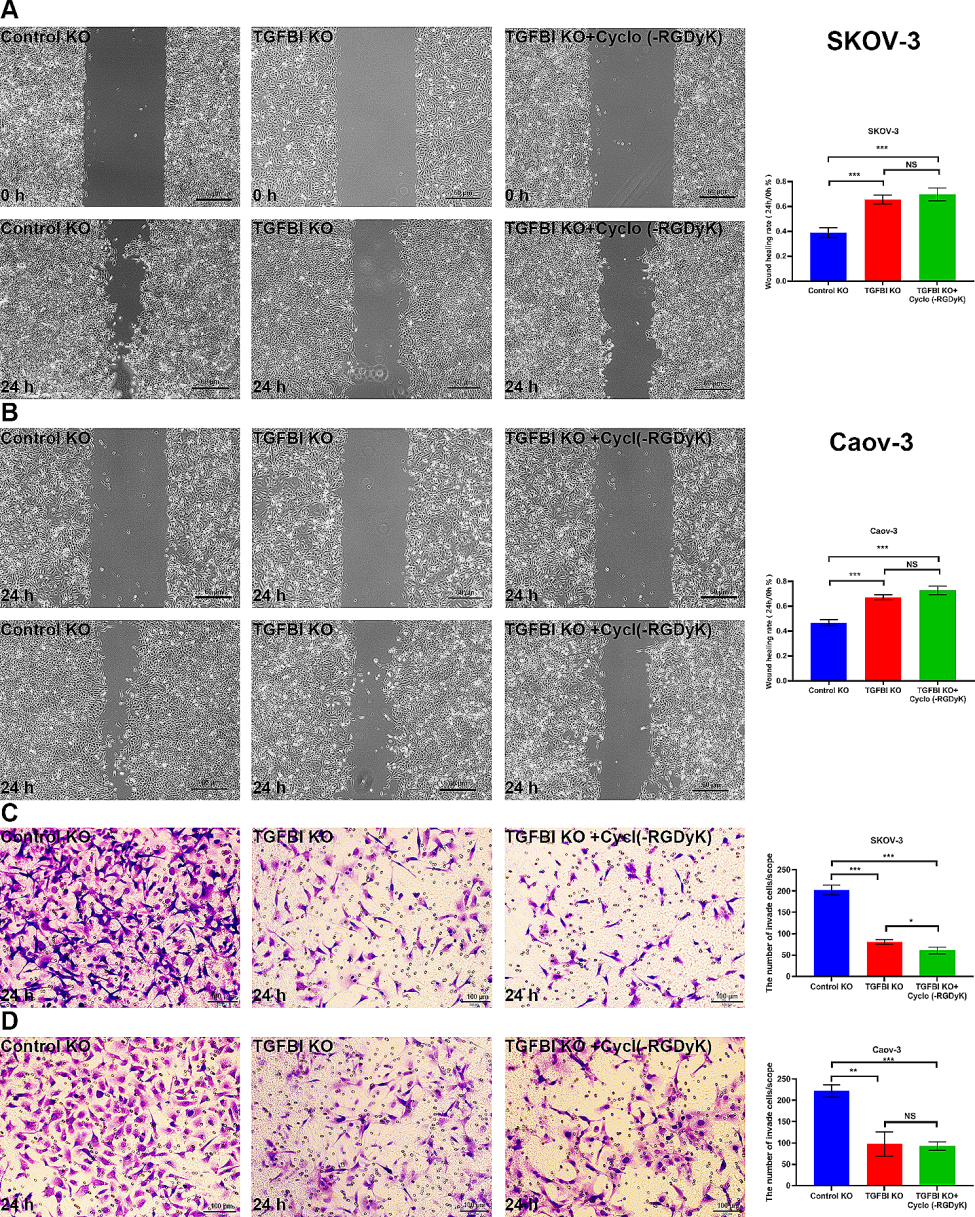
Cellular validations of TGFBI functions. **A** The changes of migratory capacity in TGFBI KO SKOV-3 cells. **B** The changes of migratory capacity in TGFBI KO Caov-3 cells. **C** The changes of invasive capacity in TGFBI KO SKOV-3 cells. **D** The changes of invasive capacity in TGFBI KO Caov-3 cells. *KO* knock out. **p < 0.01, ***p < 0.001

The functions of TGFBI were further confirmed by wound healing experiment and Transwell assay. The migration assays showed that scratches of TGFBI KO groups healed slowly and the area of cell migration significantly reduced after culture for 24 h for SKOV-3 and Caov-3 ovarian cancer cells (Fig. 5A and B). The invasive assays showed that the numbers of ovarian cancer cells that passed through the Transwell membranes were significantly decreased in the TGFBI KO groups compared to the control groups (Fig. 5C and D). These results suggested that TGFBI had essential effects on the migratory and invasive abilities of ovarian cancer cells.

### Animal validations of the TGFBI functions

**Fig. 6:**
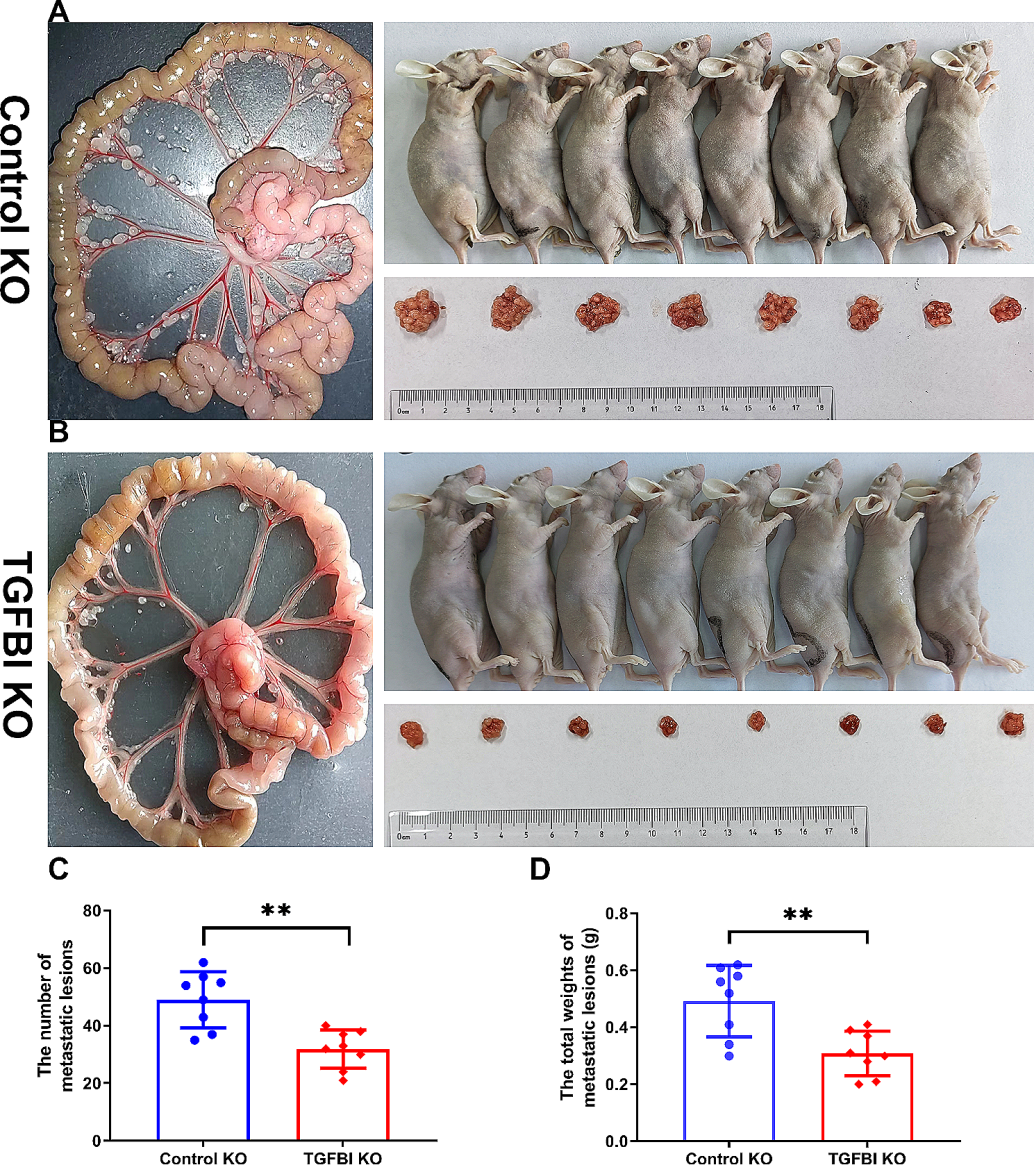
Animal validations of TGFBI functions. **A**, **B** The metastatic lesions from nude mice intraperitoneally injected with TGFBI KO SKOV-3 cell line and control cell line in gross observation. **C** The changes of the numbers of metastatic lesions from nude mice intraperitoneally injected with TGFBI KO SKOV-3 cell line. **D** The changes of total weights of metastatic lesions from nude mice intraperitoneally injected with TGFBI KO SKOV-3 cell line. *KO* knock out. **p < 0.01

Migration and invasion are closely associated with cancer cell metastasis. Therefore, we tested the effects of TGFBI on metastasis in murine peritoneal dissemination model using TGFBI KO SKOV-3 ovarian cancer cells. In the control groups, all mice showed emaciated state and cachexia, and 4 of them developed obvious ascites (Fig. 6A). However, none mice presented apparent distended abdomen in the TGFBI KO cells injection groups (Fig. 6B). Further analysis showed that there were also significant declines in the numbers and total weights of metastatic lesions in mice injected with TGFBI KO cells (Fig. 6C and D), which suggested that TGFBI affected ovarian cancer metastasis.

### Mechanisms exploration of the TGFBI functions

**Fig. 7:**
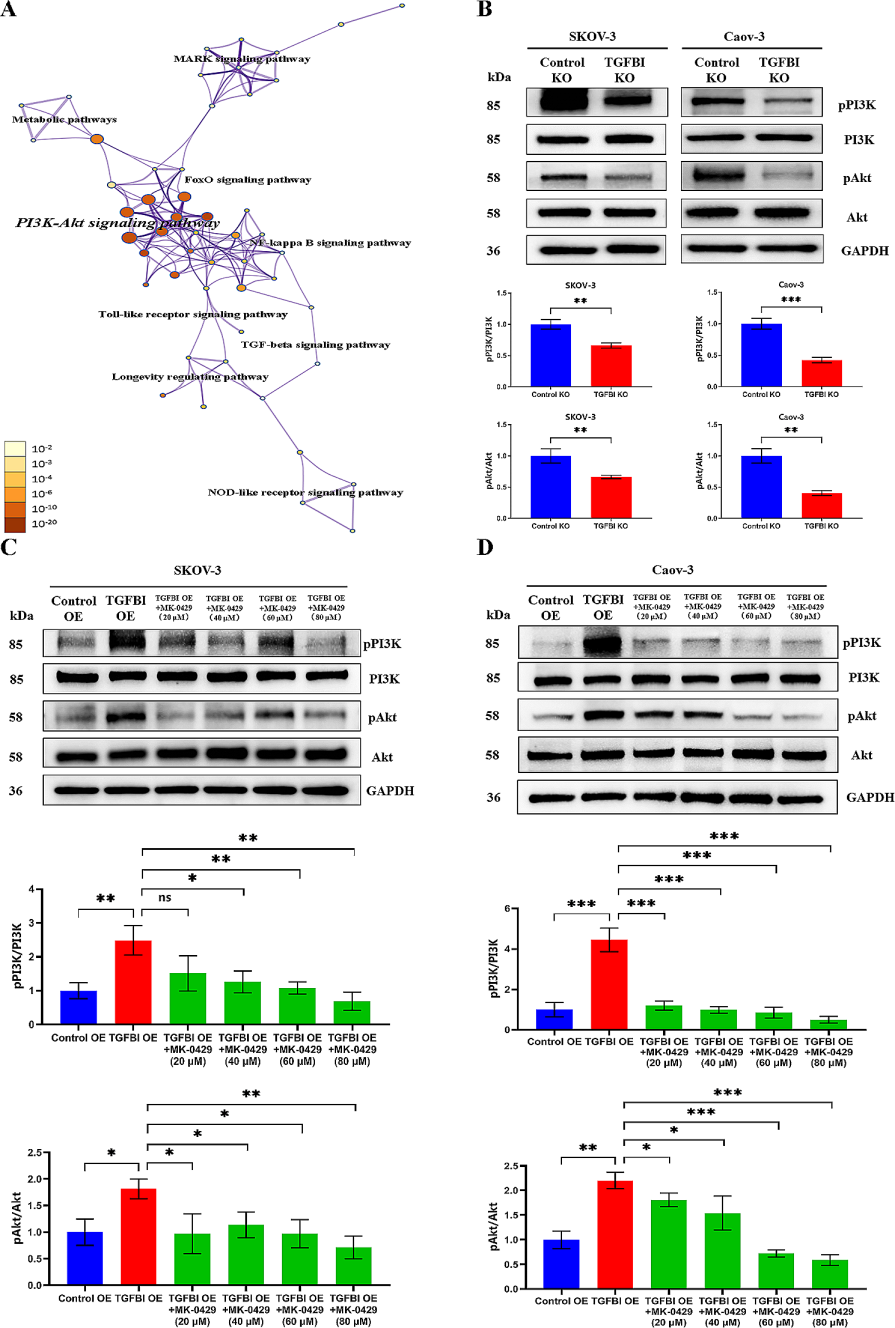
Mechanism exploration of TGFBI functions 1. **A** Proteomic analysis of the pathway enrichment in the TGFBI KO SKOV-3 cell line compared to the control cell line. **B** WB analysis of the phosphorylation levels of PI3K and Akt in TGFBI KO cell lines compared to the control ones. **C**, **D** WB analysis of TGFBI EO cell lines after treatment with broad integrin inhibitor (MK-0429). *KO* knock out, *EO* over-expression. *p < 0.05, **p < 0.01, ***p < 0.001

Next, the molecular mechanisms by which TGFBIp induced epithelial-mesenchymal transition in ovarian cancer cells were explored. Unbiased proteomics analysis of TGFBI KO ovarian cancer cells and control ones identified differential expression of multiple pathways. Among them, PI3K-Akt signaling was the most enriched pathway (Fig. 7A). Western blot analysis also confirmed that TGFBI KO ovarian cancer cells had significantly decrease levels of pPI3K and pAkt compared with control cells (Fig. 7B), while the expressions of pPI3K and pAkt were significantly increased in TGFBI over-expression cells (Fig. 7C and D). Previous studies have shown that TGFBIp regulated cell adhesion to the extracellular matrix via interacting with integrin receptors [15, 16]. Here, we found that pharmacological inhibition of integrins, which broadly targeted integrin αvβ1, αvβ3, αvβ5, αvβ6, αvβ8 and α5β1, using Integrin-IN-2 in TGFBI over-expression cells could reversed the TGFBI-induced PI3K phosphorylation and Akt phosphorylation (Fig. 7C and D). The intervention study demonstrated that TGFBIp activate PI3K-Akt signaling pathway by binding integrin receptors.

**Fig. 8:**
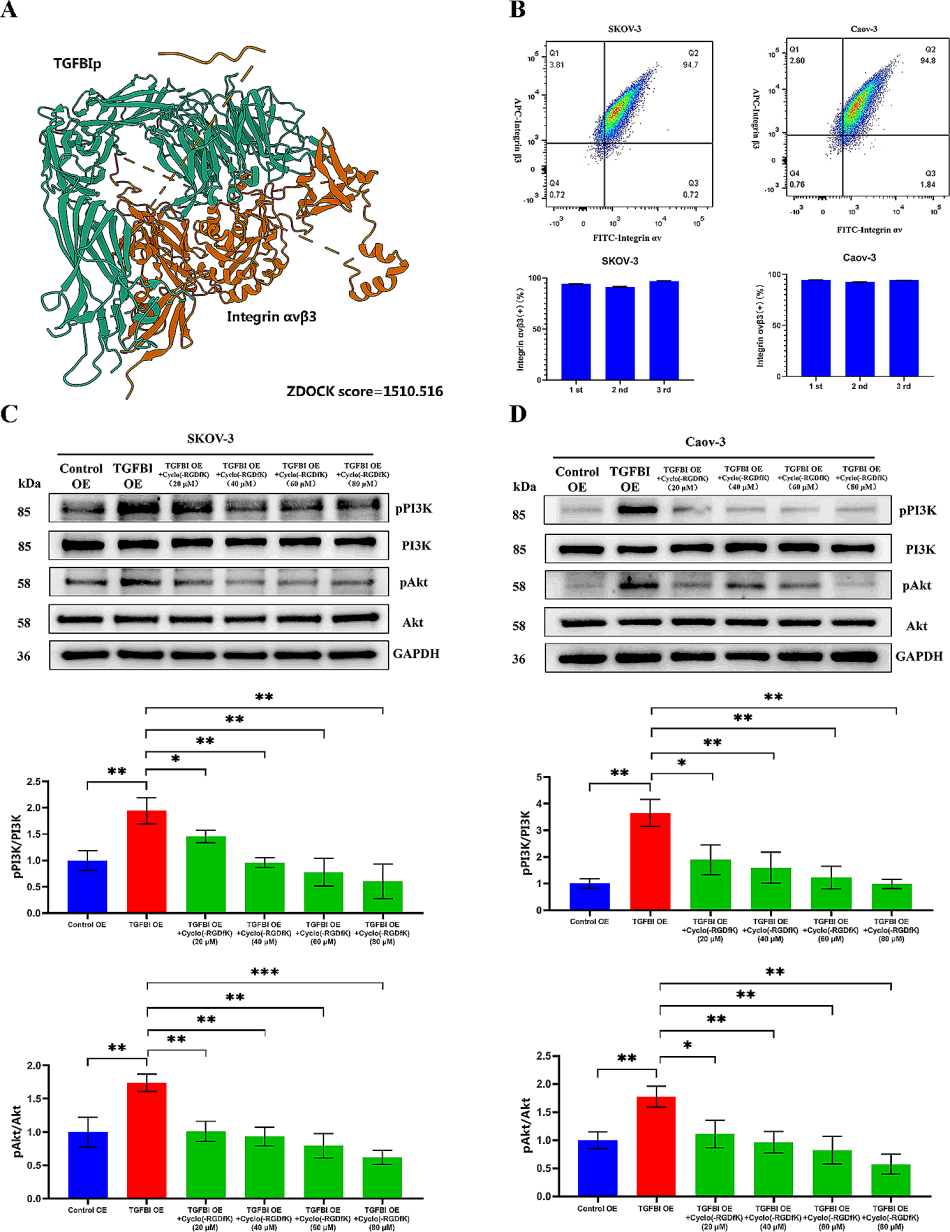
Mechanism exploration of TGFBI functions 2. **A** The molecular docking for TGFBIp and integrin αvβ3. **B** The flow cytometry analysis of the expression levels of integrin αvβ3 on the membranes of two ovarian cancer cells. **C**, **D** WB analysis of TGFBI EO cell lines after treatment with specific integrin αvβ3 inhibitor Cyclo (-RGDyK). *KO* knock out, *EO* over-expression. *p < 0.05, **p < 0.01, ***p < 0.001

The classical TGFBIp in its secreted form consists of 683 amino acids with a predicted molecular mass of 68 kDa [17]. It contains a secretory signal at the N-terminus, an EMI domain, four tandem repeat domains homologous to fasciclin I protein in Drosophila (FAS1) and a carboxy-terminal RGD sequence [17]. It was well-known that RGD peptides binded preferentially to the αvβ3 integrin, and the binding affinity of TGFBI for αvβ3 integrin was further increased due to the cooperative action of the four FAS1 domains and the RGD motif [16]. As shown in Fig. 8A, amino acid residues between TGFBIp and integrin αvβ3 formed hydrogen bonds, such as LEU531-LYS532, LEU435-ASP434 and VAL 54-GLU55, to maintain a stable protein docking model with a ZDOCK score of 1510.516. Moreover, we have shown that integrin αvβ3 was highly expressed on the surface of SKOV-3 and Caov-3 ovarian cancer cells (Fig. 8B). Therefore, we hypothesized that integrin αvβ3 might be the main targets for TGFBIp to activate PI3K-Akt signaling pathway. In the intervention study, treatment with the specific antagonist for integrin αvβ3 (Cyclo (-RGDyK)) significantly reduced PI3K phosphorylation, and subsequent pAkt levels induced by TGFBI overexpression. Together, these data demonstrate that TGFBIp activated PI3K-Akt signaling pathway in ovarian cancer cells via interacting with integrin αvβ3 (Fig. 8C and D).

**Fig. 9:**
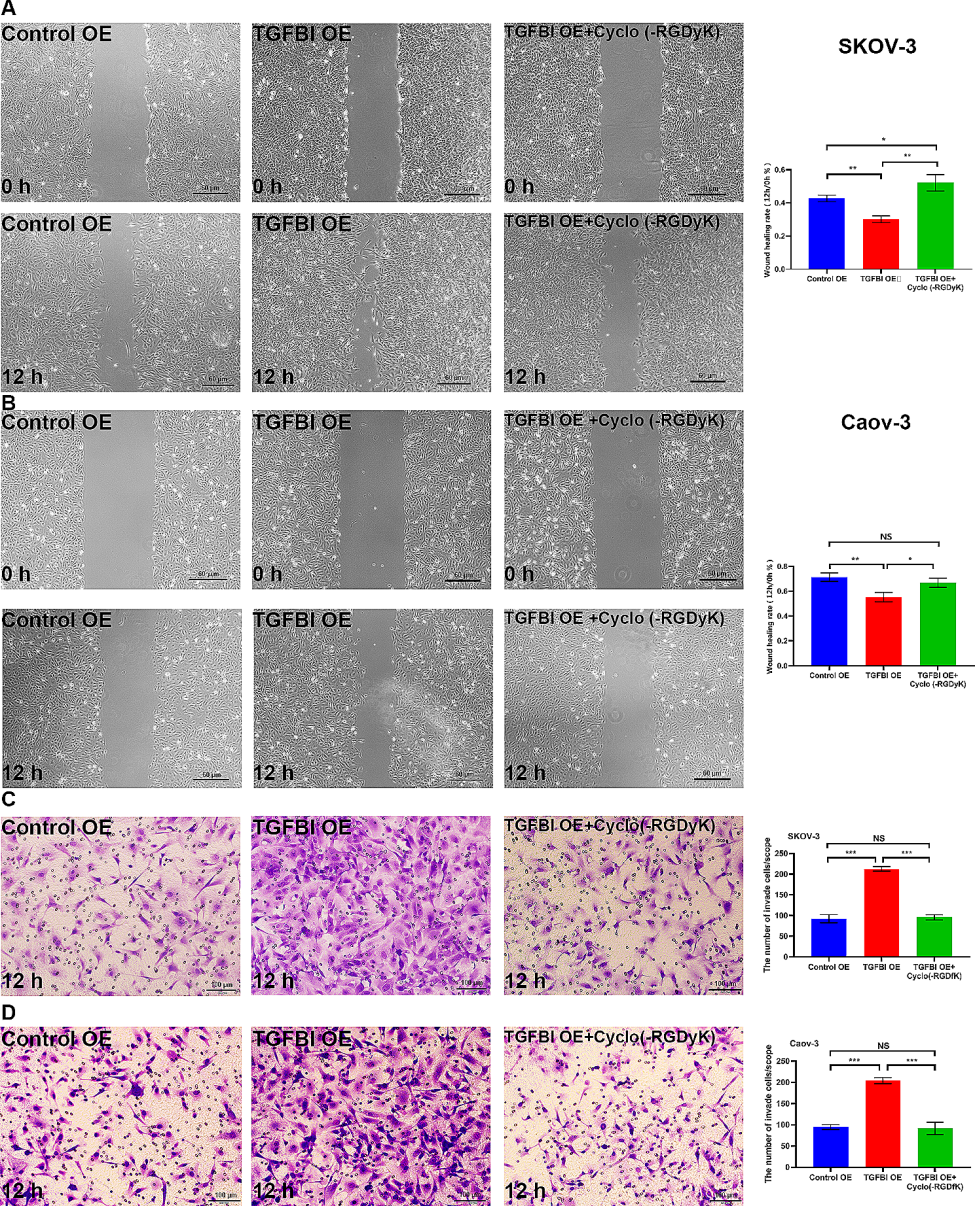
Mechanism exploration of TGFBI functions 3. **A** Wound healing assay for TGFBI EO SKOV-3 cells treated with integrin αvβ3 inhibitor Cyclo (-RGDyK) at 80 nM. **B** Wound healing assay for TGFBI EO Caov-3 cells treated with integrin αvβ3 inhibitor Cyclo (-RGDyK) at 80 nM. **C** Transwell assay for TGFBI EO SKOV-3 cells treated with integrin αvβ3 inhibitor Cyclo (-RGDyK) at 80 nM. **D** for TGFBI EO Caov-3 cells treated with integrin αvβ3 inhibitor Cyclo (-RGDyK) at 80 nM. *EO* over-expression, *NS* not significant *p < 0.05, **p < 0.01, ***p < 0.001

Moreover, we found that Cyclo (-RGDyK) could further decrease the migration and invasion of TGFBI KO ovarian cancer cells (Fig. 5). In TGFBI OE cell models, the enhanced migrate and invasive abilities empowered by TGFBI overexpressing were significantly suppressed by treatment with Cyclo (-RGDyK) (Fig. 9). Therefore, we have demonstrated the relation between migration/invasion and integrin αvβ3/PI3K/AKT activation in ovarian cancer cells.

## Discussion

In the current study, we analyzed the alteration of transcriptome profiles in the ovarian cancer cells treated with platelets, searched for new predictors for ovarian cancer and probed into their functions and mechanisms in cancer progression through bioinformatics analysis and experimental validation. As a result, we found out an adverse prognostic impact of the novel gene TGFBI on the survival of ovarian cancer patients. Moreover, TGFBI could promote metastasis by increasing migration and invasion of ovarian cancer cells via EMT induction, which might be associated with the activation of integrin αvβ3-PI3K-Akt signaling pathway.

Accumulating evidence suggests that platelets actively contribute to ovarian cancer progression. Patients with thrombocytosis were more likely to show the characteristics of aggressive malignancy in the setting of an adnexal mass at the time of diagnosis, including advance stages and high grades [9]. The poor conditions mainly resulted from the contribution of platelets to ovarian cancer metastasis. Our previous study has demonstrated that platelet treatment significantly increased the number of metastatic nodules on abdominal organs using murine models [10]. Malacrida et al. have developed an artificial tissue model consisted of mesothelial cells, fibroblasts, adipocytes, and ovarian cancer cells, and found that platelets facilitated the invasion of malignant cells into omental structure [13]. More studies, including our own, have confirmed that the invasive capacity of ovarian cancer cells could be fueled by direct exposure to activated platelets [10, 18]. Therefore, we considered platelet-treated ovarian cancer cells as the highly metastatic model to develop prognostic approaches and new therapeutics for ovarian cancer.

Finally, we identified TGFBI gene as the potential prognostic factor for ovarian cancer through a series of bioinformatics analysis. The TGFBI was firstly identified as a novel gene in the human adenocarcinoma cell line A549 after treatment with transforming growth factor-beta (TGF-β) [19].

This finding was further corroborated by the significant positive correlation between TGFBI expression and TGF-β levels in TCGA ovarian cancer samples analyzed in our study (Figure S6 A). TGFBI is located on region q31 in chromosome 5 and composed of 17 exons of almost similar sizes that span approximately 34kb [19]. The protein encoded by TGFBI is named transforming growth factor beta induced protein (TGFBIp), which comprises 683 amino acids with a predicted molecular mass of 68 kDa [17]. Structurally, TGFBIp contains a secretory signal followed by an EMI domain, four tandem consecutive FAS1 domains and a carboxy-terminal RGD motif [17]. We have detected the secreted form of TGFBIp in the culture media, and the levels changed according to the expression of TGFBI in cells. Details could be seen in the Figure S7. FAS1 domains and the RGD motif have been known to bind to integrins on the cell membranes and proteins in extracellular matrix, so the physiological functions of TGFBIp are to mediate cell to cell contacts and cell-matrix adhesion [17].

The first reported pathological manifestation related to TGFBI was corneal dystrophies due to its mutations [20]. Recently, growing evidence supported the abnormal TGFBI expression in cancer. The positive rate of TGFBIp expression was significantly higher in some tumor tissues, like colorectal cancer and esophageal carcinoma [21, 22]. Subsequently, researchers tried to employ TGFBI as a biomarker for cancer prognosis. In a study of 94 patients with oropharyngeal carcinoma, high TGFBI expression in tumor stroma was an independent prognostic factor even after statistical adjustment for age, gender, smoking status, stage, grade and treatment [23]. Cancer-specific survival was significantly better for patients with no cytoplasmic TGFBI expression in clear cell renal cell carcinoma as well [24]. We, for the first time, proposed that high TGFBI expression was associated with poor prognosis in ovarian cancer according to our bioinformatics data and IHC staining experiments (Figs. 2 and 3; Tables 5 and 1).

Although TGFBI showed controversial functions in tumorigenesis [25, 26], it was agreed that TGFBI could drive cancer progression by endowing tumor cells with highly metastatic capacity. Fico et al. crossed the TGFBI KO mouse with the MMTV-PyMT model, and illustrated that deletion of TGFBI led to a dramatic decrease stemness and invasion of breast cancer cells [27]. Ween et al. suggested TGFBIp as a promoter in peritoneal metastasis, since it could facilitate the adhesion of ovarian cancer cells to peritoneal mesothelial cells [28]. Costanza et al. found that TGFBI depletion directly reduced migration of pancreatic cancer cells [29]. In accordance with previous results, our in vitro and in vivo assays have demonstrated that TGFBI expression had impact not only on migratory and invasive capabilities of ovarian cancer cells, but also peritoneal metastatic colonization (Figs. 5 and 6). However, the concrete functions of TGFBI in molecular regulation for ovarian cancer metastasis remained unclear.

Since our bioinformatic results revealed that TGFBI expression was positively correlated with the levels of EMT markers and ECM-related genes (Figure S6 B and C), we inferred that TGFBI might be involved in EMT reprogram and ECM remodeling in ovarian cancer. In a narrow sense, EMT refers to a multiple and dynamic shift toward the mesenchymal state from epithelial phenotypes [30]. Consequently, epithelial cancer cells lose cell-cell adhesion and adopt a migratory and invasive behavior, which contribute to their shed from the primary sites and dissemination. CDH1 (E-cadherin), occludins and cytokeratins are the most commonly used markers for the epithelial trait and CDH2 (N-cadherin) and vimentin for the mesenchymal [30]. Chen et al. demonstrated that knock-down of TGFBI up-regulated the expression of CDH1 and down-regulated vimentin in RasB1 prostate cancer cells [31]. Similarly, we observed that silencing TGFBI in SKOV-3 and Caov-3 cells resulted in increased expression of CDH1 (E-cadherin) and decreased CDH2 (N-cadherin) (Fig. 4), suggesting that TGFBI affected the metastatic capabilities of ovarian cancer cells via EMT reprogram. Broadly, core EMT changes encompass basement membrane invasion and subsequent ECM remodeling, which further facilitate the progression of metastatic cascade [32]. The broken of basement membranes are mainly dependent on proteolytic ECM degradation by MMPs [33]. Ma et al. found that reduced TGFBI expression in U87 astrocytoma cells impaired their secretion of MMP-2 and MMP-9 [34]. Accordingly, we also found that TGFBI KO in SKOV3 and Caov-3 cells led to decreased expression of MMP-2 (Fig. 4), which inferred that TGFBI had the potential of remodeling ECM in ovarian cancer microenvironment.

Integrins are trans-membrane glycoprotein receptors that mediate cell adhesion and transmit signals to the cell interior [35]. Every integrin is a heterodimers of α-subunit and β-subunit, of which there are 18 and 8 variants, respectively, creating the 24 known heterodimers on the surfaces of cells [35]. Among them, overexpressed integrin αvβ3 has been found in solid cancer cells, and it has showed an important role in signal transductions of tumor proliferation and metastasis [36]. Specifically, integrin αvβ3 has the ability to cooperate with oncogenic receptor tyrosine kinases (RTKs), and then impinge on multiple pathways, including the Ras-ERK, YAP-TAZ and PI3K-Akt pathways [37]. The PI3K-Akt pathway regulates cell proliferation, growth, metabolism and motility, and is vital for normal basic cellular functions. Several studies have suggested abnormalities in the PI3K-AKT pathway are more frequent compared to other major signaling pathways in most of human cancers, including ovarian cancer [38, 39]. Aberrant activations in the PI3K-Akt signaling pathway may cause the similar endpoint: increase in tumor formation, therapy resistance, cancer cell migration and invasion [38]. It has been reported that enhanced invasive and migrate capacity in ovarian cancer are closely associated with the PI3K-Akt pathway-mediated EMT process [40]. G protein-coupled receptors (GPCRs) or RTKs interact with the regulatory p85 subunit of PI3K, triggering activation of the PI3K; Subsequently activated Akt increases the expression of EMT transcription factors, resulting in the down-regulation of the epithelial markers (CDH1), up-regulation of the mesenchymal markers (CDH2) and MMPs [40]. In our assay, we have demonstrated that TGFBI activated PI3K-AKT pathway in ovarian cancer cells by combining with integrin αvβ3 using molecular docking and inhibitors (Fig. 8). Considering its biological function of metastatic promotion, we speculated that TGFBI-induced EMT in the ovarian cancer cells might link with its ability to activate the PI3K-AKT signaling pathway.

The study surely has several limitations. Firstly, only two databases were employed for initial screen, which might result in overrepresented genes for further analysis. To solve this problem, we screened the candidate genes through multiple steps, such as overlapping DEGs identification, PPI network construction and cox proportional-hazard model analysis. Secondly, the effects of platelets were only examined using the SKOV3. It would be important to examine the effects on other cell lines including HGSOC cell models in future studies. Finally, more clinical research should be carried out to verify the prognostic effect of TGFBI on ovarian cancer.

## Conclusions

In summary, we screened out TGFBI gene in platelet-treated ovarian cancer cells through combination of our private microarray results with GEO data, and verified its prognostic value in ovarian cancer using cohorts from TCGA and tissue microarray. Hence, the expression level of TGFBI was expected to be a candidate biomarker for the construction of ovarian cancer prognostic models. Functionally, we suggested that TGFBI affect metastatic potentials of ovarian cancer cells by inducing EMT and remodeling ECM, which might be associated with the activation of integrin αvβ3-PI3K-Akt signaling pathway.

## Materials and methods

### Cell lines and culture conditions

Human ovarian cancer cell lines SKOV-3 and Caov-3 were acquired from the National Infrastructure of Cell Line Resource. The cell lines were genetically validated by STR profiling using a 20-STR amplification protocol on the ABI 3730XL Genetic Analyzer (Applied Biosystems,). Cells were grown in RPMI Medium 1640 (Gibco) containing 10% fetal bovine serum (FBS) (Nanjing SenBeiJia) and 1% Penicillin-Streptomycin (Gibco).

### Platelet preparation

#### Informed consents

were approved by the Institution Ethics Review Board for Human Studies at the Second Affiliated Hospital of Xuzhou Medical University and provided to enrolled healthy individuals. Fresh whole blood samples from healthy donors were collected in ethylene diamine tetraacetic acid (EDTA)-containing tubes and centrifuged at 100 g for 10 min. The supernatant plasma, named platelet-rich plasma (PRP), was aspirated. Platelets were then pelleted from PRP for 10 min at 1000 g, and re-suspended in RPMI Medium 1640.

### Platelet treatment

SKOV-3 ovarian cancer cells were seeded in six-well plates at a density of 2.0 × 10^5^ /well. After the cells attached to the wall, they were treated platelets prepared from 1 mL PRP (named P). Untreated cells were served as control (named C). After 48 h, the cells were harvested and washed for subsequent experiments.

### Gene microarray

Total RNA was isolated from platelet-treated SKOV-3 cells or controls (4 replicates in each group) using Trizol Reagent. The complementary DNA (cDNA) was synthesized, marked and hybridized onto GeneChip PrimeView™ Human Gene Expression Array (Affymetrix,) according to the manufacture’s protocol. The GeneChip Scanner 3000 (Affymetrix,) was used to scan the gene chip, and the data files were obtained using GeneChip Operating Software (GCOS) (Affymetrix).

### Database retrieval strategy

We systematically searched for the relevant studies on the GEO DataSets (https://www.ncbi.nlm.nih.gov/gds/). Our search strategy was shown as follow: [(“Ovarian Neoplasms”) OR (“Ovarian Neoplasm”) OR (“Ovary Neoplasms”) OR (“Ovary Neoplasm”) OR (“Ovary Cancer”) OR (“Ovary Cancers”) OR (“Ovarian Cancer”) OR (“Ovarian Cancers”) OR (“Cancer of Ovary”) OR (“Cancer of the Ovary”)] AND [(“Blood Platelets”) OR (“Blood Platelets”) OR (“Blood Platelet”) OR (“Thrombocytes”) OR (“Thrombocyte”) OR (“Platelet”)]. The inclusion criteria for the eligible studies were: (1) ovarian cancer cells were divided into two groups: one group was treated platelets, the other group was set as the control, with no added platelets; (2) RNA sequencing or gene chip were performed on the ovarian cancer cells; (3) platforms used for sequencing or gene chip were introduced. The exclusion criteria were that (1) samples contained other kinds of cells, e.g. stromal cells, besides cancer cells; (2) ovarian cancer cells and their co-incubated platelets were derived from different species; (3) the data in the downloaded files were incomplete or invalid.

### DEGs selection

The raw data expression matrices were normalized using log_2_-transformed function. The probe IDs were converted to corresponding gene symbols according to the annotation information of each platform. The DEGs analysis was achieved using the “limma” package in R language (version 4.2.1). The thresholds for DEGs identification were set as |fold change| >1.50 and p value <0.05. Volcano plots and heat maps were generated to visualize the distribution of DEGs using TBtools (version 1.049), which is a canonical toolkit developed for integrative data mining and interpretation.

### Function and pathway analysis

GO provides structured terms to describe gene product attributes based on cellular components (CC), molecular functions (MF) and biological processes (BP), while KEGG database is widely used to explore information about biological pathways involving DEGs. We used Metascape (https://metascape.org/) to perform GO annotation and KEGG pathway enrichment analysis. Metascape is an effective and efficient web-based tool designed for experimentalists to interpret large-scale datasets [41]. Minimum overlap ≥ 3 and p-value < 0.01 were considered statistically significant.

### Integration of protein-protein interaction (PPI) network analysis

Venn diagram was generated online (http://jvenn.toulouse.inra.fr/app/example.html) to present common DEGs in the included datasets. The STRING online database (version 11.5) (http://string-db.org/) was used to evaluate the potential interactions among the common DEGs. Interactive pairs with middle confidence (interaction score > 0.4) were selected to construct the PPI network using Cytoscape software (www.cytoscape.org/). Additionally, we used CytoHubba, a plugin of Cytoscape, to obtain the degree of each protein node and identify core genes, where genes of the top 20 degrees were considered as core genes.

### Prognostic and survival analysis for core genes

Firstly, the prognostic values of the core genes in patients with ovarian cancer were tested on samples from The Cancer Genome Atlas (TCGA) database by Cox proportional-hazard model. The “forestplot” package from R programming language (version 4.2.1) was applied to complete the univariate and multivariate Cox regression analysis. Core genes that showed significant hazard ratio (p value < 0.05) were considered as key genes for further analysis. The nomogram was then plotted using the “rms” package in R software (version 4.2.1) to predict the 1, 3, and 5-year overall survival. Finally, survival curve analysis was performed to validate the effect of key genes on OS, PFS and post-progression survival (PPS) of ovarian cancer patients using samples from Kaplan-Meier Plotter database (http://kmplot.com/analysis/). Key genes that significantly affected ovarian cancer prognosis were regarded as candidate genes.

### Function exploration for candidate genes

We further predicted functions for candidate genes using samples from TCGA database. The “GSVA” package in R software (version 4.2.0) was employed to present correlation between candidate genes and pathway scores by Spearman correlation, and p values <0.05 were considered statistically significant.

### Tissue microarray and immunohistochemical (IHC) staining

A human tissue microarray containing 158 carcinoma tissues from ovarian cancer patients (Lot number: T23-0025) was purchased from the Shanghai Outdo Biotech Company with ethical approval, and basic clinical information of patients was shown in the Table 5. IHC staining was performed on the tissue slide using immunohistochemical Kit (Beijing Solarbio). After deparaffinized, rehydrated in graded ethanol, and microwaved-heated in sodium citrate buffer for antigen retrieval, the slides were incubated overnight with anti-TGFBIp (Proteintech, 1:500 dilution). After washing with PBS, a horseradish peroxidase-conjugated secondary antibody was added and incubated at 37 °C for 30 min. At last, the section was stained with 3,3′-diaminobenzidine and counterstained with haematoxylin. The stained slides were assessed with immunohistochemical integrated score (IIS), which was calculated by multiplying the staining intensity score and the staining proportion score. The intensity score was determined based on the staining color: 0 for no staining, 1 for pale brown, 2 for brown, and 3 for tans. The proportion score was evaluated considering percentage of positive fields: 1 for 1–25% of fields with positive staining, 2 for 25–50% of fields with positive staining, 3 for 50–75% of fields with positive staining, and 4 for 100% of fields with positive staining.

### Lentiviral infection

The sgRNA targeting TGFBI was designed and inserted into GV392 plasmid (Shanghai GeneChem). Then the plasmid, as well as two helper plasmids, was transfected into 293T cells to construct CRISPR/Cas9 lentiviral vector. Meanwhile, TGFBI sequence was inserted into GV492 plasmid (Shanghai GeneChem) to construct over-expression (OE) lentiviral system. The virus infection assays for bulk population were performed after lentiviruses were purified and quantified. SKOV-3 and Caov-3 cells (2 × 10^6^ cells) were prepared and infected at a multiplicity of infection (MOI) of 10 with lentiviruses and corresponding controls for 24 h at 37 °C in the presence of 4 mg/ml polybrene. Stably transfected cells were obtained through puromycin selection, and efficiencies of knock-out (KO) and OE was evaluated by western blot (WB).

### Real-time quantitative reverse transcription-PCR (qRT-PCR)

Total RNA was extracted from cell samples according to the manufacturer’s instruction of Trizol reagent (Invitrogen). The reverse transcriptase kit (Takara) was used to synthesize cDNA. Quantitative real-time PCR was performed with Talent SYBR Green Kit (Beijing Tiangen) on the LightCycler 480 analyzer (Roche). The primer pair sequences of CDH1, CDH2 and MMP-2 were listed in Table S1, and GAPDH was served as a housekeeping control.

### WB

Cell samples were harvested and lysed with cold RIPA lytic buffer (Beijing Solarbio) supplemented with 1% PMSF (Beijing Solarbio) and protein phosphatase inhibitor (Beijing Solarbio). Protein concentration of cell lysate was determined using a BCA protein assay kit (Beijing Beyotime). Protein samples were loaded in equal amounts (20 µg) and separated by sodium dodecyl sulfate polyacrylamide (SDS-PAGE) gel electrophoresis (5% stacking gel at 100 V for 15 min and 10% separation gel at 130 V for 90 min). Protein blots were transferred to polyvinylidene difluoride (PVDF) membranes, and incubated in 5% skimmed milk diluted by Tris-buffered saline with 0.05% Tween 20 (TBST) for 1 h at room temperature. The membranes were incubated with primary antibodies diluted 1:1000 at 4 °C with gentle shaking overnight. After washed three times with TBST, membranes were incubated with the horseradish peroxidase (HRP)-conjugated secondary antibody (Affinity Biosciences) diluted 1:2500 for 1 h at room temperature. After the membranes were washed four times, signals of specific proteins were detected using a chemiluminescence kit (Thermo Fisher). Densitometry readings were calculated with ImageJ software. GAPDH was used as loading control. The following antibodies were used: rabbit polyclonal anti-CDH1 (Wuhan Proteintech), rabbit polyclonal anti-CDH2 (Wu han Proteintech), rabbit polyclonal anti-MMP-2 (Wuhan Proteintech), rabbit monoclonal anti-PI3K (Abcam), rabbit monoclonal anti-pPI3K (Abcam), rabbit monoclonal anti-Akt (Abcam) and rabbit monoclonal anti-pAkt (Abcam).

### Wound healing experiment

Ovarian cancer cells were seeded into 6-well plates at the density of 2.5 × 10^5^/well and cultured to 100% confluence. A 10 µl sterile pipette tip was used make a straight scratch line in the cell monolayer to create an artificial wound. Cells were then washed with PBS and suspended in the RPMI-1640 medium without FBS. Migrating cells were observed for 12 or 24 h under a light microscope. For every time point, three pictures were taken for wound gap widths measurement.

### Transwell assay

Transwell inserts (Costar) with polycarbonate membranes of 8.0-µm pore size were placed in 24-well plates (Costar). The upper chamber was coated with 80 µL mixture composed of Matrigel (Corning) and culture medium (1:7). Ovarian cancer cells were seeded into the upper chamber with serum-free medium with the amount of 1 × 10^5^. Subsequently, 600 µl complete medium was added to the lower chamber. After 12 or 24 h incubation at 37 °C, the non-invading cells on the upper surface of the filters were wiped off using cotton swabs, and the invading cells on the lower surface were fixed with methanol for 20 min and stained with 0.1% crystal violet (Solarbio,) for 5 min. Cell invasion ability was presented as the number of cells on the lower surface, and the average number of cells from three randomly selected visual fields was calculated. Results were expressed as the number of invade cells per scope.

### Animal experiments

Six-week female Balb/c Nude mice (Changzhou Cavens) were housed and maintained under specific pathogen-free conditions. The experimental protocols were approved by the Laboratory Animal Ethics Committee of the Xuzhou Medical University. Mice were divided into 2 groups (*n* = 8/group), and injected intraperitoneally with 2 × 10^6^ TGFBI KO SKOV3 cells and control cells, respectively. Four weeks later, mice were euthanized and received exploratory laparotomy. Organs in the abdominal cavity were observed and photographed for peritoneal dissemination. Metastatic lesions were harvested for count and weight.

### Mass spectrometry-based proteomic analysis

TGFBI-KO SKOV-3 ovarian cancer cells and controls were lysed with SDT buffer (4% SDS, 100mM Tris-HCl, pH 7.6). Cell lysates were homogenized by sonication and then boiled for 15 min. After centrifuged at 12,000 g for 15 min, the supernatant was quantified with the BCA Protein Assay Kit (Beyotime). Then, 150–200 µg of proteins for each sample were treated with filter-aided sample preparation (FASP Digestion), and the protein suspensions were digested with trypsin (Promega). The resulting peptides were labeled using TMT reagent according to the manufacturer’s instructions (Thermo Fisher). TMT labeled peptides were fractionated, separated and finally on a Q Exactive HF-X mass spectrometer (Thermo Fisher Scientific). MS/MS raw files were processed Proteome Discoverer 2.2 (Thermo Fisher Scientific), and searched against the protein database (Uniprot_HomoSapiens_20387_20210928_9606_swissprot). A peptide and protein false discovery rate of 1% was enforced using a reverse database search strategy. Proteins with |fold change| >1.2 and p value < 0.05 were considered to be differentially expressed proteins. Pathway analysis was performed using KEGG database.

### Molecular docking

Rigid protein-protein docking was performed between TGFBIp and Integrin αvβ3 to study the relationships. The PDB format of the protein structural domain was downloaded from the Protein Data Bank PDB database (http://www.rcsb.org/). The module was run to identify the docking sites and calculated as the ZDOCK scores.

### Flow cytometry analysis

Up to 10^6^ cells were stained with fluorochrome-conjugated antibodies for 30 min at 4 °C in the dark. The expression of integrin αv subunit and β3 subunit on the surface of SKOV-3 and Caov-3 ovarian cancer cell lines were detected with a FITC-conjugated anti-integrin αv antibody (Wuhan Proteintech) and an APC-conjugated anti-integrin β3 antibody (Wuhan Proteintech). Fluorescence was analyzed using a BD FACSCanto flow cytometer, and data were analyzed using FlowJo V10 software.

### Intervention assay

TGFBI-OE ovarian cancer cells were seeded in 5 wells of the 6-well culture plates at density of 2 × 10^5^/well and 2 × 10^6^/well, respectively. The control-OE cells were seeded in the left 1 well. The TGFBI-OE cells were treated with broad integrin inhibitor MK-0429 or specific integrin αvβ3 inhibitor Cyclo (-RGDyK) at concentration of 0 mM, 20 mM,40 mM,60 mM and 80 mM. After incubation for 48 h, cells were harvested for WB assay. For wound healing assay and Transwell assay, the concentration of Cyclo (-RGDyK) was set as 80 mM.

### ELISA assay

Culture media from TGFBI KO ovarian cancer cells, TGFBI OE ovarian cancer cells and their controls were collected after 48 h incubation. TGFBIp levels were measured using human TGFBI ELISA kit according to the manufacturer’s instructions (Abcam: ab155426).

### Immunofluorescence staining

TGFBI KO cells and their controls were fixed with 4% paraformaldehyde for 10 min at room temperature. To block the nonspecific binding, the cells were incubated with 3% bovine serum albumin (BSA) in PBS for 1 h. And then, the cells incubated with rabbit polyclonal antibodies E-Cadherin (Wuhan Proteintech) at 4 °C for 24 h. After washing with PBS three times, cells were incubated with Alexa Fluor 488-conjugated anti-rabbit IgG (Thermo Fisher) for 1 h at room temperate. Nucleic were visualized with DAPI. Images were acquired on confocal laser scanning microscope (Leica STELLARIS 5, Germany).

### Statistical analysis

The continuous variable of TGFBIp expression was divided into groups using the X-Tile software based on survival time. Kaplan-Meier analysis was used to explore the effect of intratumoural TGFBIp context and other pathological factors on survival, and log-rank test was performed for the difference between groups. Cox proportional hazard model was used to perform the univariate and multivariate analysis between risk factors and prognosis. Relative fold-change expressions of qRT-PCR were calculated by the 2^–ΔΔCt^ method. Quantification of WB and wound healing assay were performed by Image J software (National Institute of Health, Bethesda, USA). Differences between two groups of data were statistically evaluated using Statistical Package for the Social Sciences (SPSS) software 13.0 (SPSS, Chicago, USA) with Student’s unpaired t-test or Mann-Whitney U test. Data were shown as mean ± standard error, and p value < 0.05 was considered statistically significant.

### Electronic supplementary material

Below is the link to the electronic supplementary material.

Supplementary Material 1

Supplementary Material 2

Supplementary Material 3

Supplementary Material 4

Supplementary Material 5

Supplementary Material 6

Supplementary Material 7

Supplementary Material 8

Supplementary Material 9

Supplementary Material 10

## Data Availability

The datasets used and/or analyzed during the current study are available from the corresponding author on reasonable request.

## Declarations

The protocol of experiments involving tissue microarray was approved by the Affiliated Hospital of Xuzhou Medical University Ethics Committee, the Second Affiliated Hospital of Xuzhou Medical University Ethics Committee and Shanghai Qutdo Biotech Company Ethics Committee.

Oral or written informed consent was provided by all enrolled patients.

## Abbreviations

GEO: Gene Expression Omnibus
DEGs: differentially expressed genes
PPI: protein-protein interaction
IHC: immunehistochemical
OS: overall survival
PFS: progression-free survival PPS:post-progression survival
EMT: epithelial mesenchymal transition
KEGG: Kyoto Encyclopedia of Genes and Genomes
TCGA: The Cancer Genome Atlas
CC: cellular component
MF: molecular function
BP: biological process
ECM: extracellular matrix
FAS1: fasciclin I protein in Drosophila
RTKs: receptor tyrosine kinases
GPCRs: G protein-coupled receptors
ELISA: enzyme-linked immunosorbent assay
BSA: bovine serum albumin
