# Supplementary figures and images for: Novel prognostic marker TGFBI affects the migration and invasion function of ovarian cancer cells and activates the integrin αvβ3-PI3K-Akt signaling pathway
Source: J Ovarian Res. 2024 Feb 23;17:50. doi: 10.1186/s13048-024-01377-5 (PMC10885438; doi:10.1186/s13048-024-01377-5)

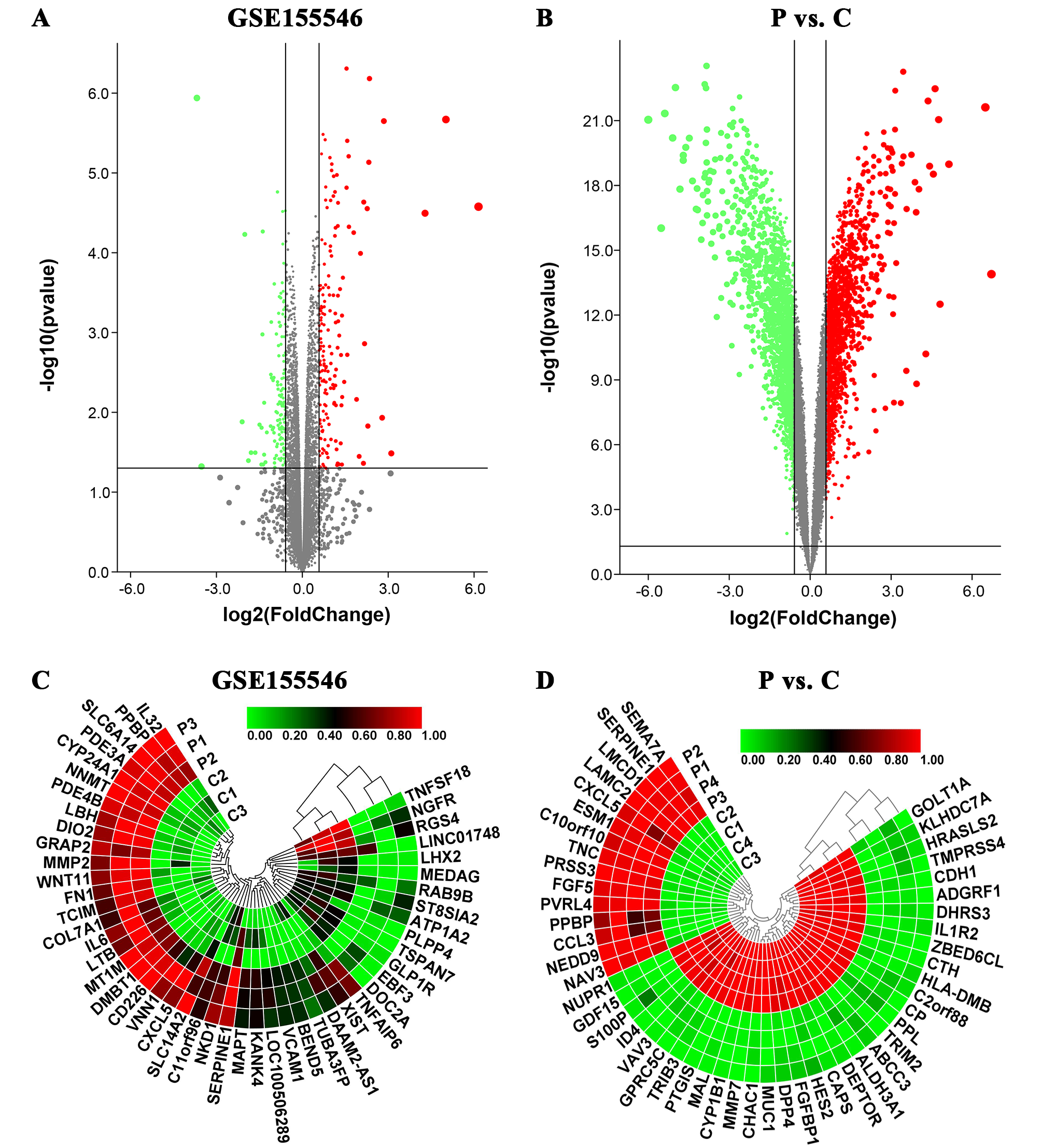

Supplement: Supplementary file 1 — Supplementary Material 1 [file 13048_2024_1377_MOESM1_ESM.tif]

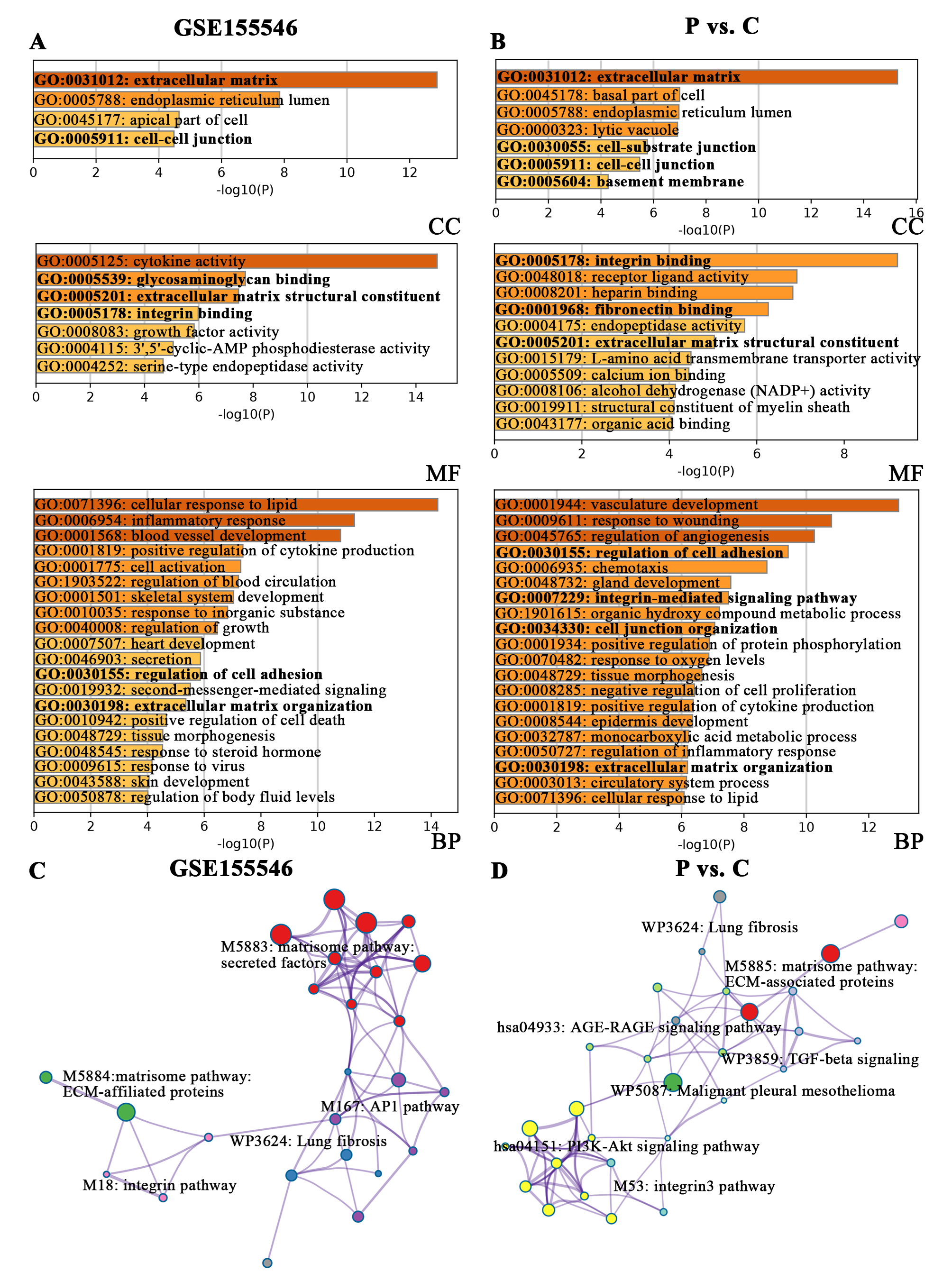

Supplement: Supplementary file 2 — Supplementary Material 2 [file 13048_2024_1377_MOESM2_ESM.tif]

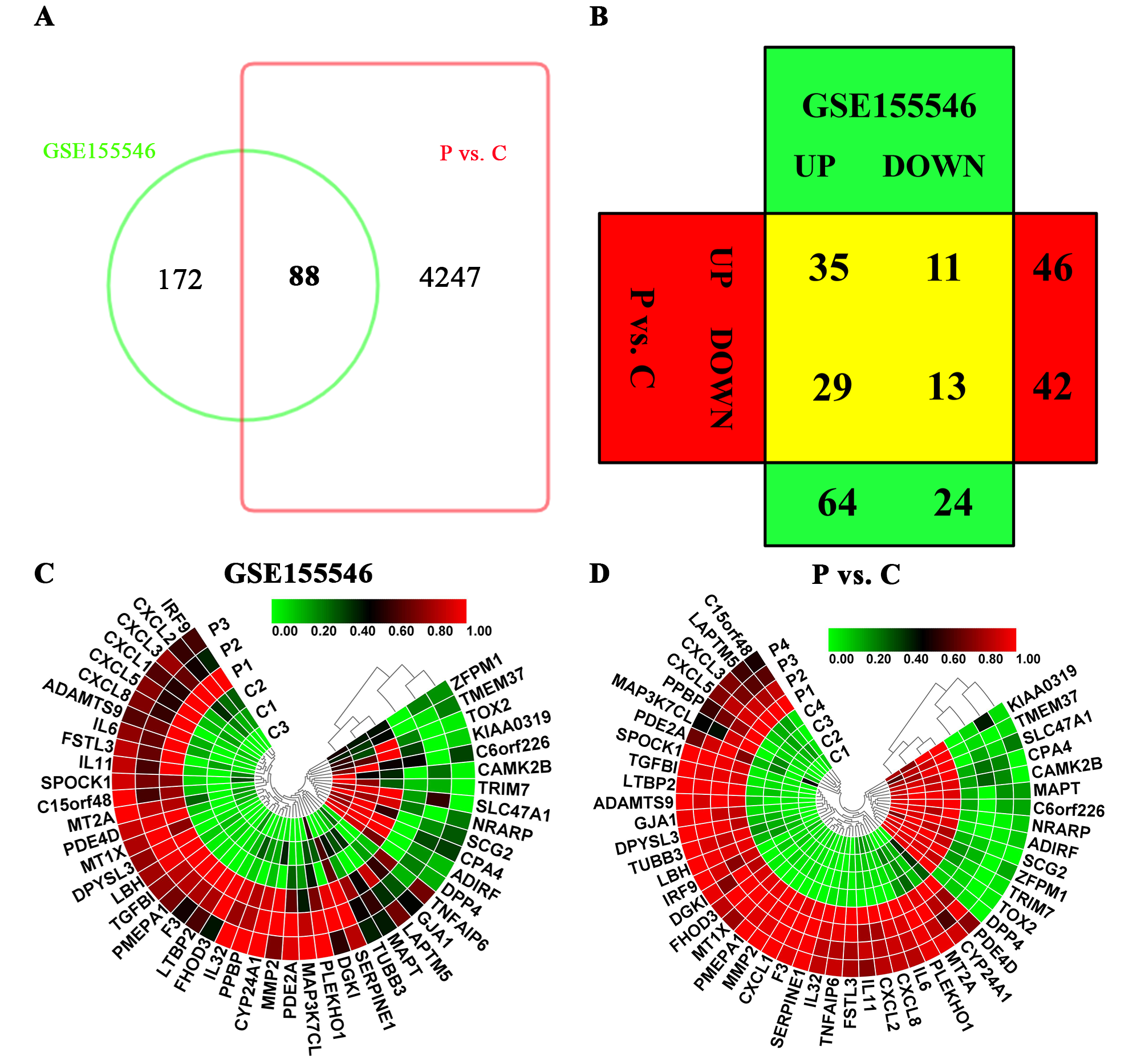

Supplement: Supplementary file 3 — Supplementary Material 3 [file 13048_2024_1377_MOESM3_ESM.tif]

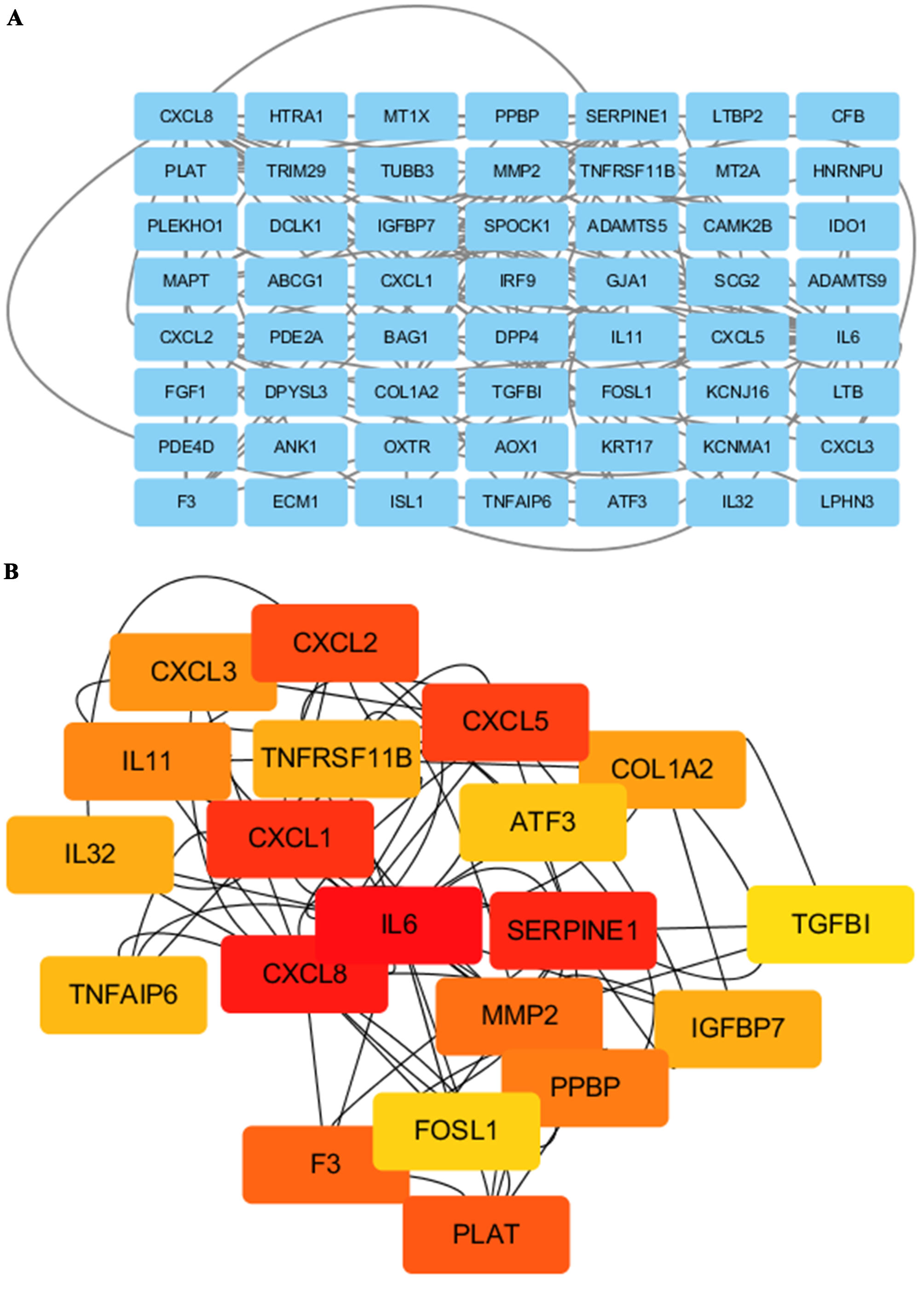

Supplement: Supplementary file 4 — Supplementary Material 4 [file 13048_2024_1377_MOESM4_ESM.tif]

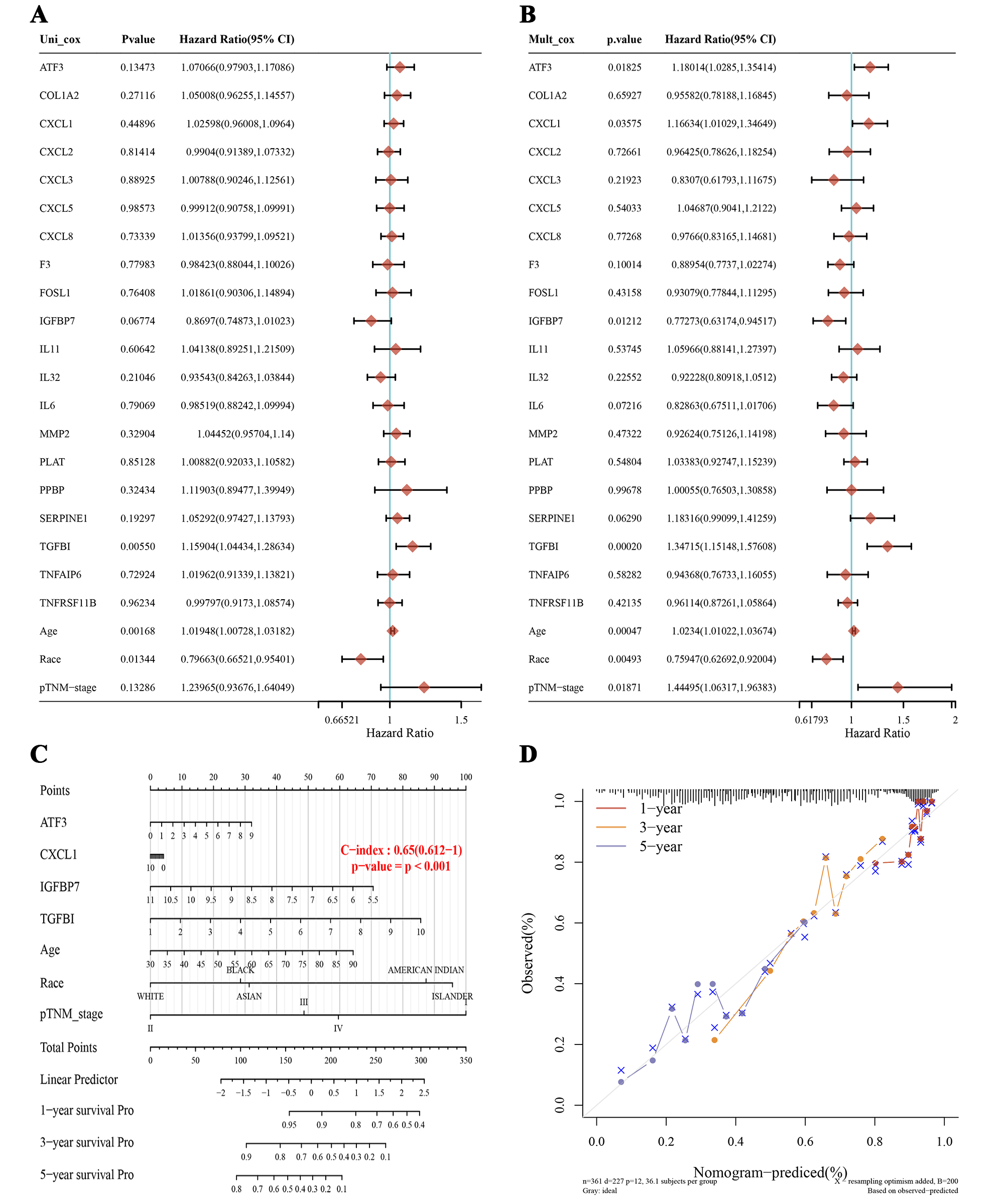

Supplement: Supplementary file 5 — Supplementary Material 5 [file 13048_2024_1377_MOESM5_ESM.tif]

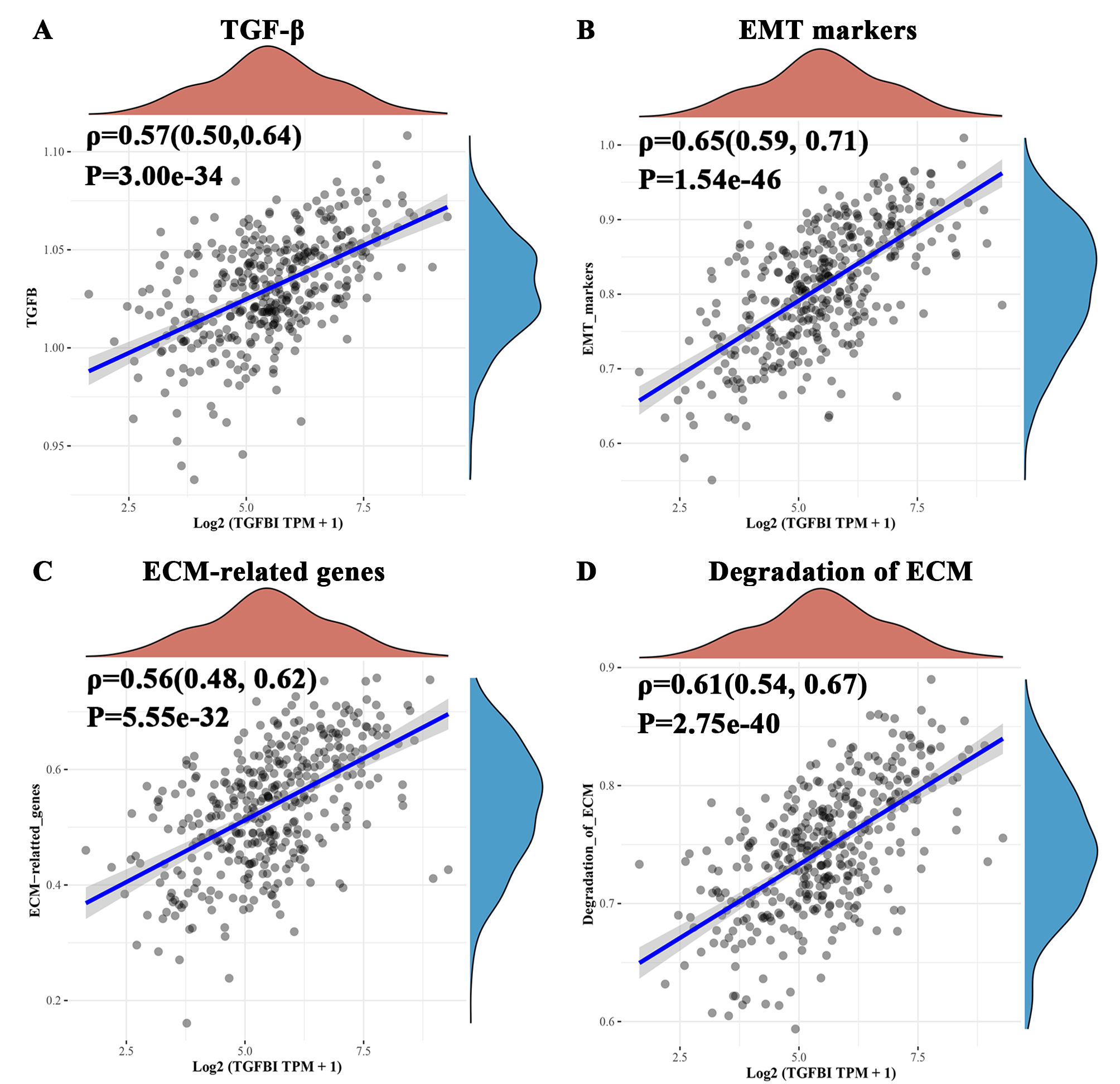

Supplement: Supplementary file 6 — Supplementary Material 6 [file 13048_2024_1377_MOESM6_ESM.tif]

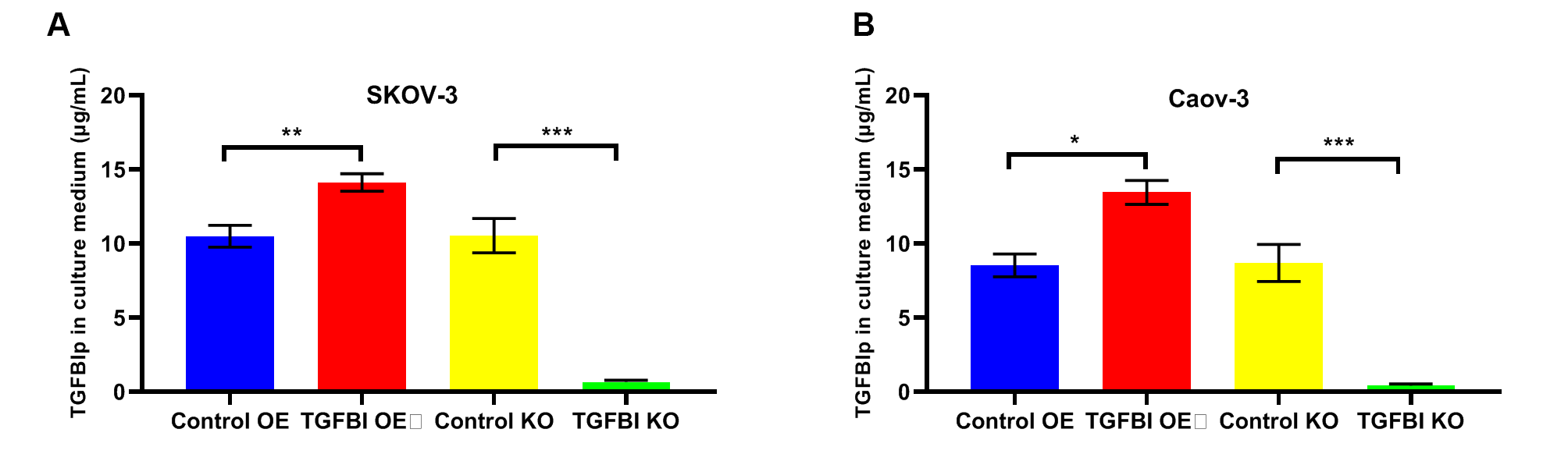

Supplement: Supplementary file 7 — Supplementary Material 7 [file 13048_2024_1377_MOESM7_ESM.tif]

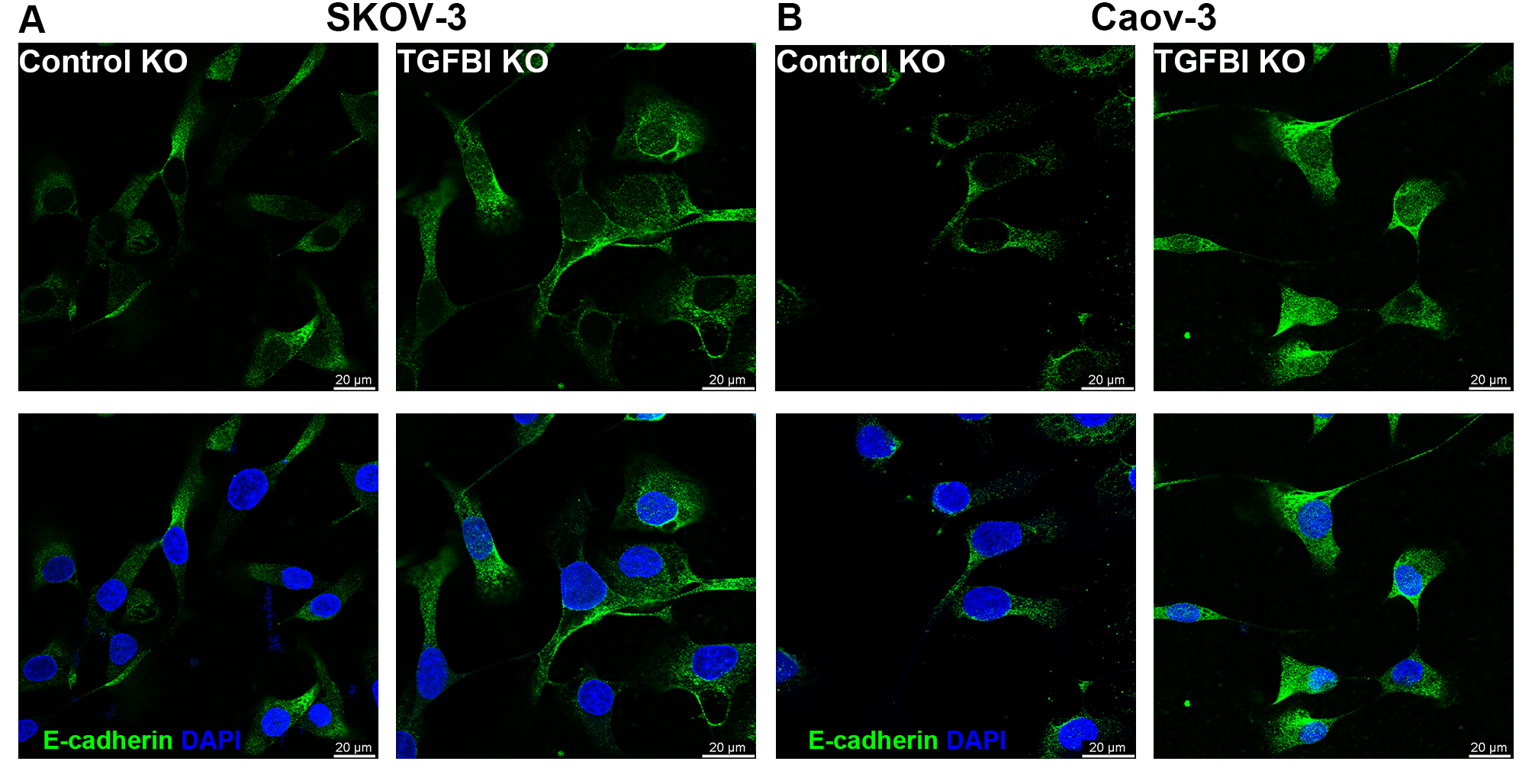

Supplement: Supplementary file 8 — Supplementary Material 8 [file 13048_2024_1377_MOESM8_ESM.tif]
