# Supplementary material for: Novel prognostic marker TGFBI affects the migration and invasion function of ovarian cancer cells and activates the integrin αvβ3-PI3K-Akt signaling pathway
Source: J Ovarian Res. 2024 Feb 23;17:50. doi: 10.1186/s13048-024-01377-5 (PMC10885438; doi:10.1186/s13048-024-01377-5)
Supplement: Supplementary file 9 — Supplementary Material 9 [file 13048_2024_1377_MOESM9_ESM.docx]

**Table S1** Sequences of primer pairs

| Gene | Sequence (5'-3') | Reference |
| --- | --- | --- |
| CDH1 | F: TGGAGGAATTCTTGCTTTGC | [1] |
| (E-cadherin) | R: CGCTCTCCTCCGAAGAAAC |  |
| CDH2 | F: ACCAGGTTTGGAATGGGACAG | [2] |
| (N-cadherin) | R: ATGTTGGGTGAAGGGGTGCTTG |  |
| MMP-2 | F: CCAACTACAACTTCTTCCCTCGC | [2] |
|  | R: GCAAAGGCATCATCCACTGTCTC |  |
| GAPDH | F: TGACAACTTTGGTATCGTGGAAGG | [2] |
|  | R: AGGGATGATGTTCTGGAGAGCC |  |

**Reference**

[2] Park J, Schwarzbauer JE: Mammary epithelial cell interactions with fibronectin stimulate epithelial-mesenchymal transition. Oncogene 2014, 33(13):1649-1657.

[1] Khan MA, Tania M, Wei C, Mei Z, Fu S, Cheng J, Xu J, Fu J: Thymoquinone inhibits cancer metastasis by downregulating TWIST1 expression to reduce epithelial to mesenchymal transition. Oncotarget 2015, 6(23):19580-19591.
